# Genome-wide identification of replication fork stalling/pausing sites and the interplay between RNA Pol II transcription and DNA replication progression

**DOI:** 10.1186/s13059-024-03278-8

**Published:** 2024-05-21

**Authors:** Patricia Rojas, Jianming Wang, Giovanni Guglielmi, Martina Mustè Sadurnì, Lucas Pavlou, Geoffrey Ho Duen Leung, Vijay Rajagopal, Fabian Spill, Marco Saponaro

**Affiliations:** 1 Institute of Cancer and Genomic Sciences, University of Birmingham, Birmingham, B15 2TT UK; 2 School of Mathematics, University of Birmingham, Edgbaston, Birmingham, B15 2TT UK; 3 Department of Biomedical Engineering, University of Melbourne, Melbourne, VIC 3010 Australia

**Keywords:** Replication fork pausing/stalling, Replication stress, DNA damage, RNA Pol II transcription, DNA replication, Transcription elongation, Replication fork speed

## Abstract

**Background:**

DNA replication progression can be affected by the presence of physical barriers like the RNA polymerases, leading to replication stress and DNA damage. Nonetheless, we do not know how transcription influences overall DNA replication progression.

**Results:**

To characterize sites where DNA replication forks stall and pause, we establish a genome-wide approach to identify them. This approach uses multiple timepoints during S-phase to identify replication fork/stalling hotspots as replication progresses through the genome. These sites are typically associated with increased DNA damage, overlapped with fragile sites and with breakpoints of rearrangements identified in cancers but do not overlap with replication origins. Overlaying these sites with a genome-wide analysis of RNA polymerase II transcription, we find that replication fork stalling/pausing sites inside genes are directly related to transcription progression and activity. Indeed, we find that slowing down transcription elongation slows down directly replication progression through genes. This indicates that transcription and replication can coexist over the same regions. Importantly, rearrangements found in cancers overlapping transcription-replication collision sites are detected in non-transformed cells and increase following treatment with ATM and ATR inhibitors. At the same time, we find instances where transcription activity favors replication progression because it reduces histone density.

**Conclusions:**

Altogether, our findings highlight how transcription and replication overlap during S-phase, with both positive and negative consequences for replication fork progression and genome stability by the coexistence of these two processes.

**Supplementary Information:**

The online version contains supplementary material available at 10.1186/s13059-024-03278-8.

## Background

Transcription allows cells to express the functional relevant parts of their genomes, while DNA replication creates two identical copies of the genome to pass on to daughter cells. Transcription and DNA replication are therefore the two essential processes for living that use the DNA as a substrate. As such, they need to be finely regulated to avoid reciprocal interference. This is particularly important for DNA replication, as impediments to replication forks progression induce DNA damage and genome instability (a condition generally referred to as replication stress). Deregulation of RNA polymerase II (RNAPII) transcription factors has been identified as a major cause of DNA damage [1]. There are several known mechanisms through which transcription can conflict with the replication machinery inducing DNA damage: (i) accumulation and persistence of RNA–DNA hybrids (R-loops [1, 2]); (ii) accumulation and persistence of topological constraints [3, 4]; (iii) accumulation and persistence of stalled/paused RNAPII (transcription stress [5, 6]). Previous evidence by immunofluorescence and microscopy suggested that transcription and DNA replication do not overlap in higher eukaryotic cells [7, 8]; as such, regions that are transcribed are not contemporary replicated, and vice versa. However, other work using similar approaches showed opposite findings [9]. In parallel, recent evidence has proposed that transcription and replication could be occupying the same regions together. Origins of replication have been identified near transcription start sites (TSSs) of long transcribed genes, with replication forks moving in the same direction of the RNAPII, reportedly to avoid head on collisions between the two machineries; moreover, ORC1 binds directly nascent RNAs to regulate replication origins activation [10–13]. Moreover, it was shown that transcription can still be active and RNAPII associated with transcribed genes even during their replication [14–16]. Nevertheless, it is still unclear what the global impact of RNAPII transcription is on DNA replication progression through genes and how this affects genome stability.

To address this problem, we analyzed replication progression with a particular focus on its advancement through genes, also establishing an assay to identify genomic hotspots where replication forks stall or pause more frequently. We found that replication stalling/pausing sites were associated with DNA damage, overlapped with previously identified fragile sites and with breakpoints of genomic rearrangements found in cancers. Importantly, short treatments with ATM and ATR inhibitors in cultured cells increased the levels of cancer-specific rearrangements. We also compared stalling/pausing sites inside transcribed regions to those in non-transcribed regions. While replication fork stalling/pausing sites in transcribed genes were directly linked to transcription progression, they presented similar DNA damage levels to stalling/pausing sites outside transcribed regions. At the same time, DNA replication progressed faster across long transcribed genes, benefitting from the reduced chromatin density produced by transcription activity. Altogether, our data highlighted the complexity of the interplay between RNAPII transcription and DNA replication, with both positive and negative implications from the coexistence of the two processes. It also identified how DNA damage response factors are important to mitigate the potential negative impact of transcription-replication collisions and avoid formation of chromosomal rearrangements.

## Results

### Identification of replication fork stalling/pausing sites

**Fig. 1:**
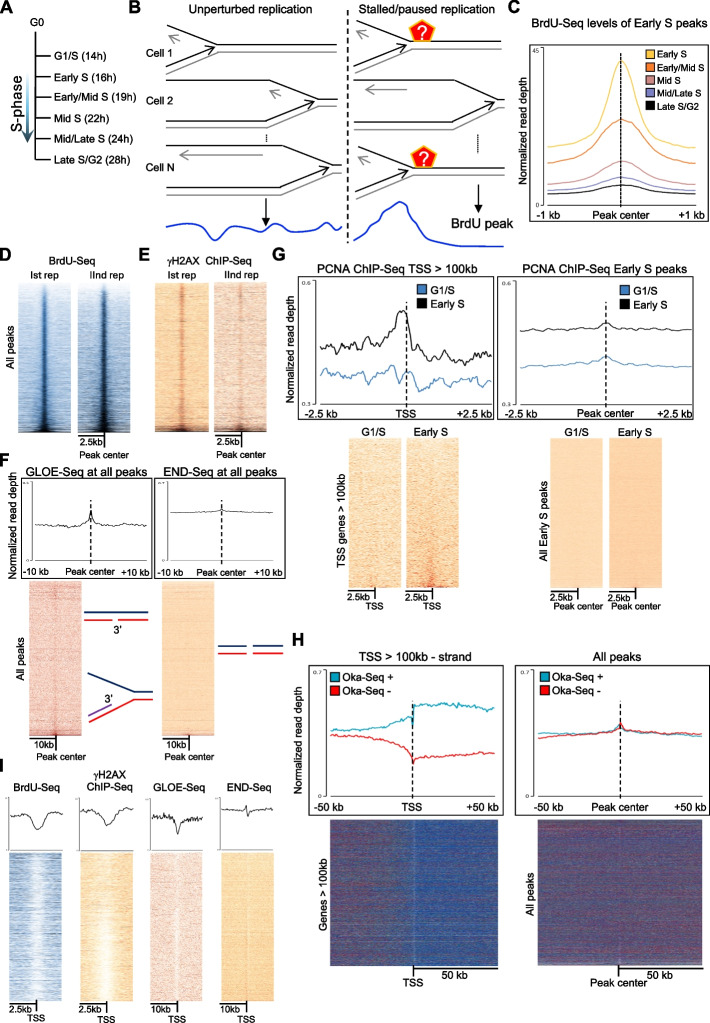
Identification of replication fork stalling/pausing hotspot sites. **A** Schematic of the kinetic of the experiment with indicated the timepoints following serum starvation: G1/S (14 h), Early S (16 h), Early/Mid S (19 h), Mid S (22 h), Mid/Late S (24 h), and Late S/G2 (28 h). **B** Schematic of the working model describing how BrdU peaks represent hotspot sites of replication forks slow down/pausing. Panel on the left: if DNA replication progresses unperturbedly through a genomic region in a cell, by averaging the BrdU-Seq signal over a population of cells, we will obtain a relatively flat profile. However, if in a specific site there is a hotspot where replication forks slow down/pause/stall, and this hotspot occurs frequently in a population of cells, when we average the BrdU-Seq signal over that region, the profile will show a peak as there is an overrepresentation of specific reads. We can computationally identify these sites performing a peak calling analysis. **C** Average metagene profile of BrdU-Seq normalized to Input DNA at the BrdU peaks called in Early S ± 1 kb in all the other timepoints. **D** Heatmap analysis of the two BrdU-Seq experiments normalized to Input DNA from [14] at all the BrdU peaks sorted by the intensity of the BrdU-Seq signal, ± 2.5 kb. **E** Heatmap analysis of γH2AX-Seq from [14] at all the BrdU peaks sorted by the intensity of the BrdU-Seq signal, ± 2.5 kb. **F** Average metagene profile and heatmap analysis of GLOE-Seq [18] and END-Seq [19] levels at all the BrdU peaks, ± 10 kb. **G** Average metagene profile and heatmap analysis of ChIP-Seq of PCNA at the Early S and G1/S timepoints at the TSS of genes > 100 kb transcribed in the Early S timepoint and at BrdU peaks identified in the first timepoint, ± 2.5 kb. **H** Average metagene profile and heatmap analysis of strand specific Oka-Seq from [12] at the “ + ” and the “–” strand, at TSS of genes > 100 kb transcribed on the “-” strand and at all the BrdU peaks, ± 50 kb. **I** Average metagene profile and heatmap analysis of BrdU-Seq, γH2AX ChIP-Seq, GLOE-Seq, and END-Seq at TSS of genes > 100 kb, ± 10 kb

Previous data in the literature evidenced how RNAPII transcription was still active during the replication of genes [14–16]. However, while interference between transcription and replication has been clearly identified in the proximity of TSSs [14, 15], nothing is known on whether transcription affected DNA replication progression through the remaining parts of genes and how this influenced genome stability. Therefore, we analyzed replication progression through genes, also establishing a procedure to identify hotspot sites where replication forks stalled and paused. The aim was to determine (i) potential determinants of where replication forks stall and pause more frequently; (ii) whether these were directly related to transcription activity; (iii) whether stalling/pausing of replication forks inside transcribed regions presented different DNA damage levels compared to stalling/pausing of forks outside transcribed regions. We analyzed previously published datasets in immortalized fibroblasts, as these monitor DNA replication throughout S-phase with multiple timepoints, allowing a precise mapping of replication progression [14]. Briefly, BJ-hTERT fibroblasts synchronized in G0/G1 by serum starvation were released in medium with serum to re-enter cell cycle, and at specific timepoints, cells were processed to analyze replication and transcription activities together (Fig. 1A [14]). To monitor DNA replication progression, cells were pulsed for 1 h before each timepoint with BrdU, followed by immunoprecipitation of the BrdU-labeled DNA and next generation sequencing (BrdU-Seq). To identify hotspots of replication fork stalling and pausing, we hypothesized that if replication forks progressed unperturbed through a region, this would lead to an even incorporation of BrdU, with the average BrdU-Seq profile across a population of cells relatively flat (Fig. 1B). When replication forks slowed down or paused at a particular hotspot, the BrdU-Seq signal would not spread. If a hotspot occurred in all cells, there would be a total decline in BrdU incorporation downstream of the replication fork stalling/pausing site; however, if a hotspot arose only in a fraction of the cells, there would be an overrepresentation of sequences where replication forks have stalled or paused, detected as an increase in BrdU signal. Accumulations of BrdU signal representing hotspots where replication forks stall/pause has been previously found in bacteria [17] and can be identified as a BrdU peak using a bioinformatic peak calling procedure (Fig. 1B).

We identified BrdU peaks in each timepoint, finding 33,295 BrdU peaks in total throughout the genome. The highest number of BrdU peaks was found in the first timepoint, with 17,869 peaks (Early S, 16 h after serum starvation release). BrdU peaks were transient, evidenced comparing the BrdU-Seq signal over the peaks in each timepoint to the following one (Fig. 1C, Additional file 1: Fig. S1A); this indicated that most replication forks stalling/pausing events were generally short-lived and can be identified only when assessing replication progression with multiple timepoints throughout S-phase. BrdU peaks hotspots were also reproducible and consistent across two repeats (Fig. 1D). We postulated that if BrdU peaks represented indeed replication forks stalling and pausing sites, they would be associated with features present at replication forks stalling and pausing, like increased DNA damage, presence of replication fork stalling/pausing associated factors and exposed 3′-ends. Hence, we performed a ChIP-Seq of phospho-Ser139 H2AX (hereafter γH2AX) in asynchronous cells as a proxy for DNA damage across all cell cycle stages [14, 20–23]. We found enrichment for γH2AX across BrdU peaks as evidenced by the heatmap analysis (Fig. 1E). To exclude that BrdU peaks represented conversely sites with increased DNA synthesis because of DNA damage repair activities, we also performed a γH2AX peak calling analysis, to assess how much DNA damage repair-induced DNA synthesis would affect BrdU levels. We observed increased BrdU levels at the γH2AX peaks; however, these were much lower than those found at BrdU peaks that at the same time presented lower γH2AX levels than γH2AX peaks. Therefore, while we cannot exclude that part of the BrdU increase observed at the BrdU peaks might be consequence of DNA damage repair synthesis, BrdU levels at γH2AX peaks are not as high as at BrdU peaks (Additional file 1: Fig. S1B).

Next, we analyzed published datasets for double strand specific markers 53BP1, XRCC4, and Ligase IV (AsiSI-ER-U2OS cells [24, 25]) and replication stress-associated factors BRCA1 (MCF10A cells [26]), BRCA2 (hTERT-HME1 cells [27]), FANCD2 (U2OS cells [28]), and RAD51 (K562 cells, https://www.encodeproject.org). BrdU peaks appeared not associated with double strand breaks, as both average profiles and heatmaps showed no enrichment for 53BP1, XRCC4, and Ligase IV (Additional file 1: Fig. S1C). We observed instead an enrichment for replication stress associate factors at BrdU peaks (Additional file 1: Fig. S1C). Finally, we analyzed GLOE-Seq (which maps 3′-ends, i.e., single strand breaks and stalled replication forks) [18] and END-Seq (which maps double strand breaks) datasets [19] (Fig. 1F). In agreement with the previous findings, GLOE-Seq signal was enriched at BrdU peaks, while END-Seq signal was not (Fig. 1F, Additional file 1: Fig. S1D). Altogether, our BrdU peak calling procedure identified transient sites of accumulation of replication forks, associated with increased DNA damage levels, accumulation of replication stress-associated factors, and exposed 3′-ends.

### BrdU peaks overall did not correlate with previously identified replication origins

Previous work used peaks of modified nucleotides incorporation to identify replication origins [29–31]. Although the overall peak calling procedure was similar, there were important experimental differences between our approach and those used in the above-mentioned papers: (i) our analysis used multiple timepoints throughout S-phase as peaks were transient (Fig. 1C, Additional file 1: Fig. S1A); (ii) we pulsed cells with BrdU for longer periods; hence, the BrdU-Seq signal spread further away from replication origins. To avoid confusion on what our BrdU peaks represented, we validated that BrdU peaks identified in our experimental set up were not origins of replication. Firstly, we performed a ChIP-Seq of PCNA in G1 and Early S timepoints in BJ-hTERT cells. In eukaryotic cells, PCNA is detectable at origins of replication before the signal reduces when replication forks move away, and PCNA is unloaded from forks at sites of replication stress [32, 33]. We identified a small enrichment for PCNA in the Early S timepoint at the TSSs of long genes, previously shown to be hotspots for replication origins [12], but not at BrdU peaks (Fig. 1G). Similarly, we found an enrichment for the replication origin binding factors Orc1 and Orc2 [34, 35] at TSSs of long genes, but not at BrdU peaks (Additional file 1: Fig. S1E). Next, we analyzed whether BrdU peaks were functionally behaving like origins, analyzing Okazaki sequencing (Oka-Seq) data from hTERT immortalized fibroblasts [12], similar to the BJ-hTERT we used. Oka-seq is strand specific and identifies replication origins because of the split in the signal of Okazaki fragments being all on the Watson or the Crick DNA strand on either side of a replication origin [12]. At TSSs of long genes, we identified such a separation of Oka-Seq levels depending on the direction of gene transcription (Fig. 1H); however, this separation was not present at BrdU peaks, independently of the timepoint (Fig. 1H, Additional file 1: Fig. S1F). Equally, BrdU-Seq, γH2AX ChIP-Seq, GLOE-Seq, and END-Seq heatmaps and metagene profiles were different at the TSSs of long genes compared to BrdU peaks (Fig. 1I). Regarding the recruitment of the replication stress-related factors BRCA1, BRCA2, FANCD2, and RAD51, there was only a clear recruitment of BRCA1 and BRCA2 at the TSS of long genes (Additional file 1: Fig. S1G), in agreement with their roles in the regulation of RNAPII transcription [26, 27]. This result indicated that BrdU peaks were passively replicated and not hotspots for replication origins.

Next, we assessed the overlap between our BrdU peaks with replication origins identified using different assays: SNS-Seq detecting highly ubiquitous origins present across many cell lines and stochastic origins present only in specific cell lines [36], Ini-Seq from bladder cells [29], and Oka-Seq from fibroblasts [37, 38]. Each approach had different sensitivities regarding the number of identified origins and their size (SNS-Seq: total origins 320,748, median 0.24 kb; Ini-Seq: total origins 25,054, median 1.2 kb; Oka-Seq: total origins 12,482, median 20 kb). We found that thousands of BrdU peaks overlapped with regions identified in the different studies as replication origins (Additional file 1: Fig. S1H). Distances between peaks and annotated origins averaged from 2.7 kb against the > 250,000 stochastic origins identified by SNS-Seq up to 54 kb against origins identified by Oka-Seq (Additional file 1: Fig. S1H). However, with the exception of BrdU peaks overlapping with Ini-Seq sites, we did not observe enrichment for PCNA at BrdU peaks overlapping with origins (Additional file 1: Fig. S1I) nor we observed a separation between the Watson and Crick strand for Oka-Seq levels at peaks overlapping with identified origins, like we found at TSSs of long genes (Additional file 1: Fig. S1L). This indicated that even though many of the regions we have identified as BrdU peaks overlapped with previously identified origins of replication, for the vast majority, these regions were not active as replication origins in our cells.

Next, we analyzed further genomic features around BrdU peaks. Regions underlying the BrdU peaks had an average AT content of 59.15%, in line with the average AT content of the human genome [39], supported by the high frequencies of nucleotide hexamers with an even AT/GC nucleotide content (Additional file 1: Fig. S2A), different from the high GC content found at origins [36]. Finally, ChIP-Seq for histone H3 in asynchronous cells to assess chromatin organization around the BrdU peaks found the presence of a nucleosome positioned at the center of the peak (Additional file 1: Fig. S2B). However, except for H3K27 acetylation at the BrdU peak sites in the latest timepoint, there was no general enrichment for any of the histone modifications we analyzed (Additional file 1: Fig. S2C).

### Replication fork stalling/pausing sites inside transcribed genes were linked to transcription progression

**Fig. 2:**
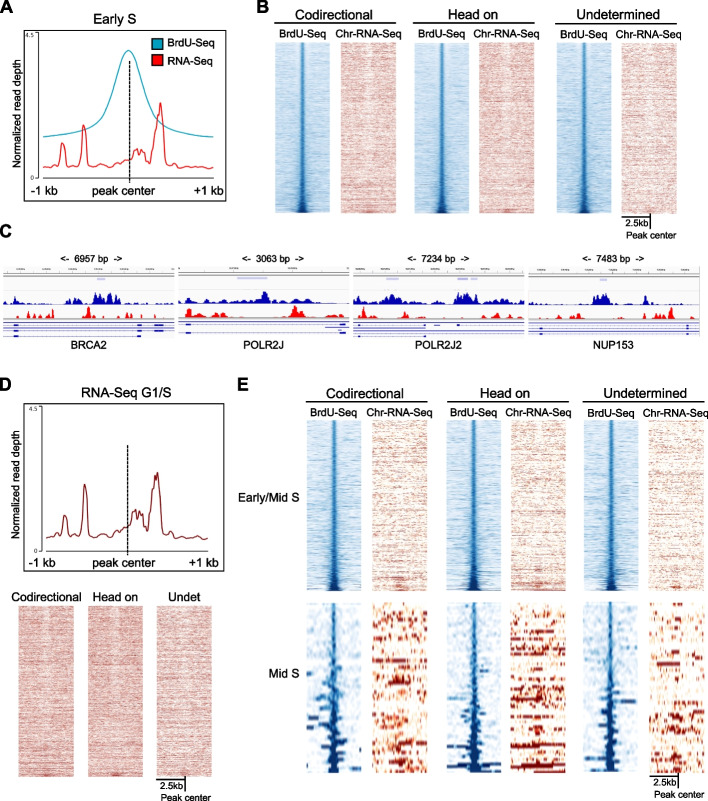
Correlation between DNA replication and transcription progressions. **A** Average metagene profiles of BrdU-Seq (blue) and Chr-RNA-Seq (red) levels from the Early S timepoint at BrdU peaks called in Early S ± 1 kb. **B** Heatmap analysis of BrdU-Seq (blue) and Chr-RNA Seq (red) signal across BrdU peaks called in Early S ± 2.5 kb, depending on the reciprocal directionality between DNA replication leading fork and transcription in codirectional, head on or undetermined, ranked by BrdU-Seq signal intensity. **C** BrdU-Seq (blue) and Chr-RNA-Seq (red) profiles across specific BrdU peaks, indicated by the light blue bar in the listed genes, with indicated the width of the region showed. **D** Average metagene profile of Chr-RNA-Seq levels and heatmap analysis from the G1/S timepoint across all the BrdU peaks called in Early S ± 1 kb, separated by directionality; Undet = undetermined. **E** As **B** but for BrdU peaks called in Early/Mid S and Mid S

Once we identified replication fork stalling/pausing sites genome wide, we determined whether those inside transcribed regions were directly associated with transcription activity, aiming to identify transcription-replication collision sites. To do this, we analyzed strand specific chromatin-bound RNA-Seq, that included also nascent RNA, to determine transcription activity at the same timepoints of the BrdU-Seq samples from [14]. Of the 17,869 BrdU peaks found the first timepoint, 6971 BrdU peaks were inside a transcribed gene. Using previously established reciprocal directions between transcription and replication for each gene [14], we found similar distribution of BrdU peaks in genes where transcription and replication moved in the same direction (codirectional, 34.83%) or in opposite direction (head on, 35.17%); the remaining was over genes where replication entered the gene from both ends (undetermined, 30%). When we analyzed the Chr-RNA-Seq profile around BrdU peaks, we observed enrichments for nascent transcription surrounding the peak center (Fig. 2A). This was further supported by the heatmap analysis at BrdU peaks that showed an overall reduction in transcription activity where the BrdU peak was identified, independently of the reciprocal directionality, as evident at single BrdU peak examples (Fig. 2B, C). When we analyzed Chr-RNA-Seq levels at BrdU peaks in a strand specific manner, we could still see this accumulation of nascent RNA on both sides of the BrdU peak by heatmap and average profiles (Additional file 1: Fig. S3A). This suggested that replication forks stalling/pausing occurred encased within two sites of accumulation of transcription. Our analyses showed similar profiles for transcription in G1/S (i.e., before replication) and for Early S (i.e., during replication) (Fig. 2D). This suggested that transcription and replication progressions were linked together and that DNA replication would adapt to established patterns of transcription progression, including where RNAPII accumulate and not vice versa. To assess whether the BrdU-Seq and Chr-RNA-Seq patterns observed were related or coincidental because of the reciprocal distributions of the signals, we performed two further analyses. First, we randomly shuffled BrdU peaks identified in the first timepoint within the same genes in which they occurred, to assess whether this generated a Chr-RNA-Seq distribution around the peaks like the one observed. Shuffling of peaks changed the levels of BrdU signal and also that of the Chr-RNA-Seq (Additional file 1: Fig. S3B). Next, we assessed whether there was direct correlation between the read counts of the BrdU-Seq and Chr-RNA-Seq across a 1-kb window. The presence of a strong positive correlation would indicate that BrdU-Seq and Chr-RNA-Seq signals were generally close together. We found no correlation between the BrdU-Seq and Chr-RNA-Seq reads counts, with similar results when performing the analysis in reverse (Additional file 1: Fig. S3C). This would indicate that the distributions of Chr-RNA-Seq and BrdU-Seq reads were independent of each other and the fact that we found a BrdU-peak close to a Chr-RNA-Seq accumulation signal was specific of the sites where it happened. As a control, we also analyzed the correlation between BrdU-Seq and γH2AX ChIP-Seq that indicated that accumulations of BrdU signal and DNA damage occur in general in close proximity (Additional file 1: Fig. S3C).

To validate that the Chr-RNA Seq signal was representative of RNAPII accumulation, we performed a ChIP-Seq of total RNAPII in the G1/S and Early S timepoints. We found that RNAPII accumulated around the BrdU peak sites in the same way in both timepoints, even though the ChIP-Seq had a lower resolution compared to the Chr-RNA-Seq signal (Additional file 1: Fig. S3D). Previous work found that RNAPII pauses at the 3′-end of exons to support splicing of transcripts [40, 41]. Therefore, we assessed whether BrdU peaks were linked to splicing-dependent pausing sites, calculating the distance between BrdU peaks and their nearest exon. The average distance between BrdU peaks and exons was approximately 10 kb in the early timepoints and up to approximately 50 kb in the last timepoint (Additional file 1: Fig. S3E), indicating that BrdU peaks were not associated in general with RNAPII pausing at exons. Therefore, these RNAPII accumulations represented bona fide hotspots of RNAPII stalling/pausing [5], suggesting that replication fork stalling/pausing hotspots inside genes occurred within two close sites of RNAPII stalling/pausing and enriched in longer genes (Additional file 1: Fig. S3F and data not shown).

Finally, we analyzed at the Chr-RNA-Seq signal around BrdU peaks in all other timepoints. We found that also BrdU peaks identified in Early/Mid S showed an enrichment of transcription activity around the BrdU peak (Fig. 2E). At later timepoints, however, this enrichment was less evident (Fig. 2E) because replication traveled through genes that were less transcribed (Additional file 1: Fig. S3G).

### Transcription elongation directly affected replication progression through genes

**Fig. 3:**
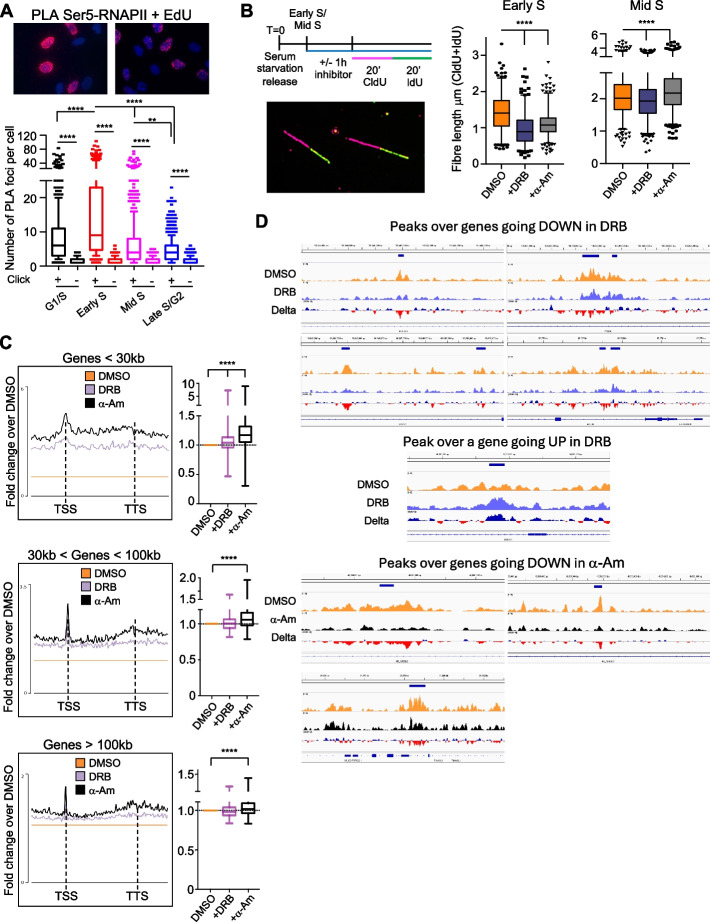
Transcription elongation affects DNA replication rates. **A** Proximity ligation assay (PLA) with cells treated with EdU and anti-Ser5-P-RNAPII antibody; representative images with DAPI staining in blue for nuclei and PLA foci in red; cells are synchronized as Fig. 1A and harvested at the indicated timepoints after 10 min incubation with EdU. PLA foci count in cells with $\ge$ 1 PLA foci (right panel), box-whisker plots 10–90 percentile with line at the median, Mann–Whitney *t*-test. *n* = 3, > 150 cells counted per timepoint in each repeat. **B** Schematic of the experiment with a representative image of DNA fibers; cells pretreated for 1 h with DRB (100 μM) or α-amanitin (α-Am, 3 μg/ml) before each timepoint as in Fig. 1A, followed by 20 min pulse with CldU and 20 min pulse of IdU; DNA fiber lengths measured using ImageJ; *n* = 3, > 400 fibers analyzed per condition; box whiskers plots with line at the median 2.5–97.5 percentile, Mann–Whitney *t*-test. **C** Average metagene profiles of BrdU-Seq from TSS to transcription termination site (TTS) for transcribed genes specifically replicated across in the first timepoint clustered by gene length, in cells treated for 1 h with DMSO, DRB (100 μM), or α-amanitin (α-Am, 3 μg/ml) before the Early S timepoint while being pulsed with BrdU, with quantifications of the BrdU-Seq signals from TSS to TTS in the DRB or α-amanitin samples as fold changes normalized to DMSO; paired nonparametric *t*-test. **D** IGV snapshot over BrdU peaks (highlighted by the blue bar) in genes replicated in the first timepoint that change expression levels following treatment with DRB or α-Am. BrdU-Seq profiles in DMSO (orange), α-Am (black), and DRB (blue) treated cells; delta represents the combination of the DRB or α-Am track with the DMSO one (DRB—DMSO or α-Am—DMSO), with blue signal indicating higher BrdU-Seq levels in DRB or α-Am compared to DMSO and red indicating higher BrdU-Seq levels in DMSO compared to the transcription inhibitors. * ≥ *p*-value < 0.05, ** ≥ *p*-value < 0.01, **** ≥ *p*-value < 0.0001

Our previous data evidenced that transcription and replication progressions might be linked together especially in early S-phase and that they might be overlapping over the same genomic regions. As such, we hypothesized that transcription and replication could be close enough to interact together and that changes to transcription elongation rates could directly change DNA replication rates too. A previous study showed that RNAPII interacts with DNA polymerases in human cells [42]. However, we were unable to reproduce these data when samples were treated with the DNase/RNase Benzonase, indicating that the two machineries might be adjacent on the DNA rather than directly interacting together (data not shown). Therefore, we performed a proximity ligation assay (PLA), which generated a positive signal only if the two factors of interest were within 40 nm of distance. Cells were pulsed for 10 min with the nucleotide analog 5-ethynyl-2′-deoxyuridine (EdU) using EdU ClickIt to label actively replicating DNA as in [43] and an anti-Ser5-RNAPII antibody for transcriptionally engaged RNAPII (Fig. 3A). In agreement with our hypothesis and our previous data, the number of cells with Ser5-RNAPII-EdU PLA foci was highest in the Early S timepoint, progressively reducing later during S-phase (Fig. 3A). These data therefore suggested that active transcription can be re-established on newly replicated DNA within the 10 min pulse of the EdU, similar to what was previously shown [16].

Next, we assessed the impact of transcription elongation inhibitors on DNA replication progression. We used alpha-amanitin (α-Am), a cyclic peptide that traps RNAPII in a conformation that reduces its ability to incorporate new nucleotides and leads to RNAPII degradation [44], and 5,6-dichloro-1-beta-D-ribofuranosylbenzimidazole (DRB), a CDK9 inhibitor that halts newly initiated RNAPII near the TSS and the transcription termination site [5, 45]. We measured how much α-Am and DRB reduced transcription elongation rates with a single gene analysis on the SPTAN1 gene. Cells were treated for 3.5 h with DRB to accumulate RNAPII near the TSS [5, 46]. After DRB removal and wash out, RNAPII restarted transcribing synchronously the SPTAN1 gene; 20 min later, we added DMSO, DRB, or α-Am to monitor RNAPII progression along the gene with different timepoints and primers at increasing distances from the TSS (Additional file 1: Fig. S4A). Elongation rates were calculated based on the time needed to reach the further distant primers in control DMSO or transcription inhibitor-treated cells. These analyses showed that DRB and α-Am reduced elongation rates over the SPTAN1 gene by 52% and 33% (Additional file 1: Fig. S4A). We then used DNA fiber analysis to measure replication fork speeds at the Early S and Mid S timepoints with or without transcription inhibitors. We hypothesized a greater impact of DRB and α-Am at the beginning of the S-phase when transcription and replication greatly overlap, rather than later in S-phase. Cells were treated with DMSO, DRB, or α-Am for 1 h before each timepoint to slow down transcription elongation and released in the nucleotides analogs chlorodeoxyuridine (CldU) and iododeoxyuridine (IdU) for 20 min each to label ongoing replication (Fig. 3B). Following stretching and denaturation of the DNA, we measured the length of the CldU/IdU-labeled fibers. We observed faster replication rates at the Mid S timepoint compared to the Early S one as previously published [47, 48]. Importantly, transcription elongation inhibitors decreased replication fork rates specifically in Early S phase (Fig. 3B). Moreover, this slowdown was progressive, as the length of the IdU label was also shorter than the CldU one (Additional file 1: Fig. S4B).

Prolonged treatment with transcription inhibitors could affect the expression of replication factors that are specifically transcribed in S-phase [49] and activate the p53 pathway because of global mRNA synthesis inhibition [50]. Therefore, we performed mRNA-Seq to assess the impact of DRB and α-Am on gene expressions and whether the observed effect on replication fork rates could be an indirect consequence of gene expression deregulations. Cells were treated as for the DNA fiber experiments followed by RNA extraction and mRNA sequencing. DRB had a stronger impact on gene expression compared to α-Am at both timepoints, with hundreds of genes showing a > 2-fold change (FC) decrease compared to DMSO (Additional file 1: Fig. S4C). Importantly, there was no enrichment in the gene ontology analysis for replication factors nor of any specific factor previously shown to be related to DNA replication (Additional file 2: Table S1). Moreover, there was a significant overlap in the genes affected at both timepoints. Similarly, there was no induction of p53 targets such as p21 and PUMA, previously shown upregulated following prolonged treatments with DRB (Additional file 1: Fig. S4D). We also analyzed the expression of histones genes by RT-PCR, as these are not assessed in a poly-A mRNA-Seq because not poly-adenylated, finding no general defect in histones expression (Additional file 1: Fig. S4E). Altogether, these data indicated that the replication forks slowdown was likely a direct effect due to replication and transcription machineries being co-present on genes, rather than an indirect consequence due to the downregulation of specific replication factors or histone genes.

To investigate the impact of transcription inhibitors on replication genome-wide, we performed a BrdU-Seq experiment in Early S, pulsing cells with BrdU in parallel with DMSO, DRB, or α-Am. Treatments with DRB and α-Am overall increased BrdU-Seq signal in genes, both visible with average metagene profiles and gene to gene FC compared to DMSO (Fig. 3C). This indicated increased occupancy and persistence of replication across genes, indicative of slower replication progression, and was more evident over shorter higher transcribed genes (Fig. 3C, Additional file 1: Fig. S4F). Importantly, this replication progression was transcriptionally dependent, as for example it was not present at not transcribed genes > 100 kb replicated in the first timepoint (Additional file 1: Fig. S4G). The fact that this phenotype was most evident over shorter genes excluded a general DNA replication defect due to the downregulation of specific transcripts because of the transcription inhibitors, as this would show increased BrdU-Seq signal in all genes to the same extent. We analyzed also whether treatments with DRB and α-Am affected the frequency of DNA replication pausing at the BrdU peaks, but the result was negative (Additional file 1: Fig. S4H). This would indicate that DRB and α-Am induced a general slowdown of replication progression across transcribed genes but not an increase in replication fork stalling/pausing. Nevertheless, we observed numerous BrdU peaks increasing or decreasing BrdU-Seq signal, depending on whether the genes were up- or down-regulated following treatment with the transcription inhibitors. This further links changes in genes transcription directly to replication progression through such genes (Fig. 3D).

### BrdU peaks were hotspots of genome instability also in cancers

**Fig. 4:**
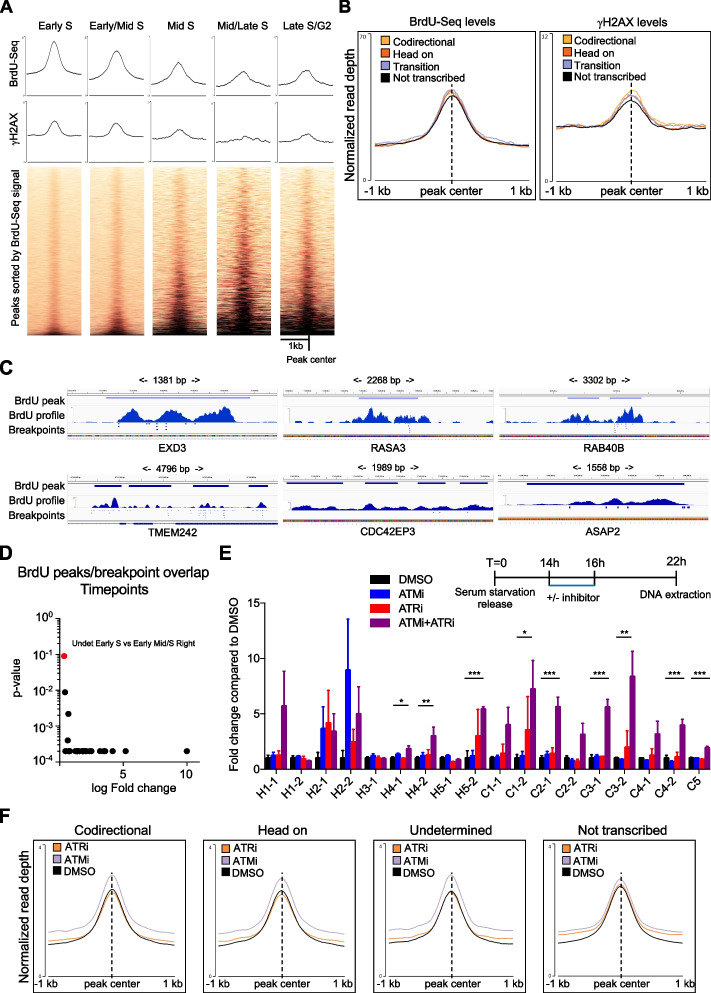
BrdU peaks are hotspot of genome instability and rearrangements at transcription-replication collision sites require ATM and ATR. **A** Average metagene profile and heatmap analysis for γH2AX at BrdU peaks identified in all timepoints, sorted by BrdU-seq levels as Fig. 1D. **B** Average metagene profile for BrdU levels normalized to Input DNA and γH2AX levels at peaks called in Early S, separated depending on the specific location of the peak. **C** Single BrdU peak examples overlapping with breakpoints of rearrangements from Cosmic website. **D** Comparison of the frequency of increased overlap of BrdU peaks called in the first timepoint with breakpoints towards all the other timepoints measured in fold change with relative permutation *p*-value, analyzing separately the left (L) and the right (R) breakpoint for each rearrangement; highlighted in red is the only comparison that is not statistically significantly different. **E** Fold change compared to DMSO of detection of specific rearrangements by RT-PCR, following treatment in Early S for 2 h with ATMi (10 μM), ATRi (4 μM), or ATMi and ATRi together; *n* $\ge$ 4, average mean ± SEM, two-sided Student *t*-test. **F** Average metagene profile for BrdU levels normalized to Input DNA at BrdU peaks in Early S separated in codirectional, head on, undetermined or not transcribed; cells treated as in **D** with BrdU added in the last hour. * ≥ *p*-value < 0.05 ** ≥ *p*-value < 0.01, *** ≥ *p*-value < 0.001

Because BrdU peaks presented overall increased DNA damage levels (Fig. 1E), we examined the link between genome instability and BrdU peaks further. Analyzing γH2AX levels at peaks in all timepoints, we found that γH2AX levels were generally higher at peaks identified in the first two timepoints, correlating with higher BrdU-Seq levels (Fig. 4A). Next, we assessed whether DNA damage levels were different at transcription associated BrdU peaks compared to BrdU peaks outside transcribed genes. We found that BrdU-Seq levels at these peaks were similar, indicating that replication fork stalling/pausing on average was not particularly affected by a specific subtype of peak (Fig. 4B). Equally, analyzing γH2AX levels we found only small differences between transcribed versus not transcribed regions (Fig. 4B). This indicated that independently of what caused replication forks stalling/pausing, there are only minor differences on how these stalling/pausing sites are associated with increased genome instability. R-loops have been identified as an important source of replication stress and genome instability [1, 2]. Therefore, we analyzed R-loops ChIP-Seq (DRIP-Seq) data from fibroblasts [51] and U2OS cells [52] at BrdU peaks sites, finding however no correlation to our BrdU peaks (Additional file 1: Fig. S5A).

Subsequent, we analyzed whether BrdU peaks overlapped with genomic hotspots of DNA damage. We analyzed the overlap with replication fragile sites, regions prone to instability following inhibition of DNA synthesis, finding significant overlap between BrdU peaks in the first timepoints with common fragile sites (CFS [53]) and early replicating fragile sites (ERFS [54]) (Additional file 1: Fig. S5B). In agreement, genes in CFS and ERFS were preferentially replicated at the beginning of the S-phase (Additional file 1: Fig. S5C). Next, we determined whether BrdU peaks were potentially linked to hotspots of genome instability in diseases, analyzing overlap with breakpoints of rearrangements found in cancer patients. To increase the number of events analyzed in our study, both breakpoints of a rearrangement were analyzed as an independent event. We found many BrdU peaks overlapping with breakpoints, with several BrdU peaks hotspots for many breakpoints (Fig. 4C, D). To determine whether this overlap was significant, we compared the frequency of peaks overlapping with breakpoint to that of a random distribution of events, using a bootstrap permutation analysis. Initially, we analyzed whether replication timing affected the overlap between BrdU peaks and breakpoints, comparing BrdU peaks identified in the first timepoint against those identified all other timepoints. We almost invariably found that BrdU peaks in the first timepoint overlapped more frequently than in other timepoints, independently of the specific subtype (Fig. 4D, Additional file 3: Table S2). This showed that replication fork stalling/pausing sites in early S phase overlapped more with sites of rearrangements in cancer cells, in agreement with the higher BrdU-Seq and γH2AX-Seq levels and overlap with fragile sites. Next, we analyzed whether the reciprocal direction of transcription and replication affected the frequency of overlap between breakpoints and BrdU peaks, finding very little difference between peaks in codirectional or head on genes and only in the latest timepoints where the number of BrdU peaks was low (Additional file 1: Fig. S5D, Additional file 3: Table S2). Intriguingly, we found that BrdU peaks in not transcribed regions were overlapping more with breakpoints than BrdU peaks in transcribed sites (Additional file 1: Fig. S5E, Additional file 3: Table S2). This could indicate that rearrangements involving not transcribed regions might be maintained with higher frequency in cancer cells and/or highlight differences in the way DNA damages are repaired. Previously, the DNA damage checkpoint kinases ATM and ATR were found activated following collisions between replication forks and R-loops, in a directional manner [55]. Therefore, we tested whether ATM and ATR prevented the formation of rearrangements at transcription-replication collision sites, designing PCR primers for 18 randomly selected rearrangements overlapping BrdU peaks in transcribed regions, at either codirectional or head-to-head sites. Following short treatment with ATM or ATR inhibitor, the RT-PCR levels of the rearrangements were normalized to the DMSO treated sample, to normalize for rearrangement events already present in our established cell line. Short-term inhibition of ATM or ATR was sufficient to induce a slight increase in rearrangements at some of the sites (Fig. 4E). When we combined the two inhibitors together, we observed however a greater increase in rearrangements affecting most of the events, independently of directionality (Fig. 4E). This suggested that both ATM and ATR were important to stabilize replication forks at transcription-replication collision sites and/or limited the formation of rearrangements. To analyze this further, we performed a BrdU-Seq in Early S treating cells with ATM or ATR inhibitors to determine whether inhibition of DNA checkpoint kinases affected BrdU-Seq levels at peaks, indicating a role in regulating how frequently replication forks pause/stall at these sites, or whether ATM and ATR are more important in regulating how pausing/stalling events are repaired. We observed a slight increase of BrdU signal with the ATM inhibitor, more evident at BrdU peaks in transcribed regions than in not transcribed regions (Fig. 4F). This would suggest that inhibition of ATM may increase overall the likelihood of a replication fork stalling/pausing. In the case of ATR, we did not identify global changes to BrdU-Seq levels at peaks; hence, it may be more important to protect stalled forks from collapse and/or controlling repair pathways to avoid the formation of a rearrangement (Fig. 4F). At the same time, we found also instances where BrdU-Seq levels at peaks changed specifically in genes whose expression was affected by ATR inhibition [56] (Additional file 1: Fig. S5F).

Finally, we analyzed whether DRB and α-Am could affect the formation of rearrangements, because of their influence on replication fork progression (Fig. 3B, C). Cells in Early S were treated with DRB or α-Am similarly to the ATM and ATR inhibitors, but neither increased rearrangement frequencies, with few cases where the transcription inhibitors reduced the formation of rearrangements below the DMSO control treated sample (Additional file 1: Fig. S5F). This agreed with the fact that DRB and α-Am do not affect replication fork stalling/pausing at BrdU peaks (Additional file 1: Fig. S5G).

Altogether, these results evidenced that replication fork stalling/pausing sites were hotspots for genome instability and overlapped with sites of rearrangements found in cancer cells. Moreover, they determined ATM and ATR important in mitigating the formation of rearrangements at transcription-replication collision sites, working together and separately for this role.

### Replication progressed faster along long transcribed genes

**Fig. 5:**
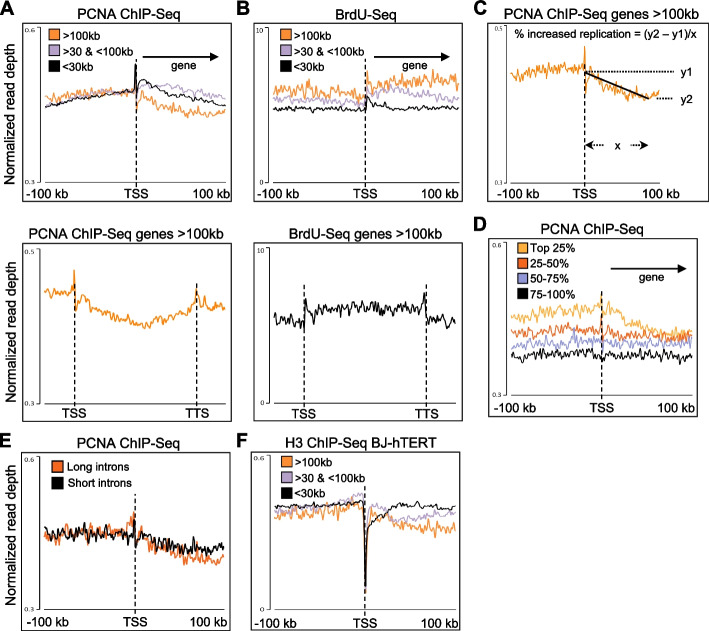
Transcription-dependent increased DNA replication rates across long genes. **A** Average metagene profile for PCNA ChIP-Seq at transcribed genes replicated in the first timepoint clustered by gene length, ± 100 kb around the TSS; bottom panel from TSS to TTS only for genes > 100 kb. **B** As for **A** with BrdU-Seq from the first timepoint. **C** Schematic on how the increase in replication rates across the gene length has been calculated. **D** Average metagene profile for PCNA ChIP-Seq at genes > 100 kb, separated in four groups based on their transcription levels from [14] Chr-RNA-Seq, TSS ± 100b. **E** As for **A** but for genes > 100 kb ranked according to the length of their first intron in long (116 genes) or short introns (116 genes). **F** As for **A** but with ChIP-Seq of histone H3

Previously, we found that changes to transcription elongation impacted differently on the replication of genes depending on gene length (Fig. 3C). Transcription elongation rates are not constant from gene to gene, and generally RNAPII transcribes faster on long genes because it needs long introns and reduced exon density to reach full speed [57]. Therefore, we aimed to determine whether transcription elongates rates could had a general influence on how fast replication progressed through genes. In a ChIP-Seq experiment, the levels of replication fork components are highest at origins and then reduce as replication forks move away and spread. Consequently, the faster forks move, the lower the signal of replication fork components is. Hence, we decided to use our PCNA ChIP-Seq data to have a relative measure of replication fork rates through genes (i.e., high PCNA levels—> lower speed, low PCNA levels—> higher speed) and identify changes in replication rates across different regions. When we analyzed the PCNA ChIP-Seq signal at genes clustered by gene length, we found that upstream of the TSS and PCNA levels were the same independently of gene length (Fig. 5A). However, with the increase of the gene length, we observed a progressive reduction in PCNA ChIP-Seq signal, with the lowest point approximately 75 kb after the TSS for genes > 100 kb (Fig. 5A). This was not due to a lack of replication of these regions, as the BrdU-Seq signal across the same regions was unaffected (Fig. 5B). To determine whether these changes in ChIP-Seq densities were specific to PCNA, we analyzed MCM7 ChIP-Seq data from HeLa cells [58], finding that also in this case progressively decreased levels correlating with gene length (Additional file 1: Fig. S6A). Next, to determine how much faster replication progressed along genes from the TSS to lowest point density point, we measured the rate of reduction of PCNA and MCM7 ChIP-Seq densities along genes longer than 100 kb. We found similar values, with a 17.2% reduction for PCNA (Fig. 5C) and 16.7% for MCM7 (Additional file 1: Fig. S6B). The decrease in ChIP-Seq densities correlated directly with transcription activity, as PCNA ChIP-Seq was clearly reduced only in the top 25% most transcribed long genes (Fig. 5D), and MCM7 ChIP-Seq signal reduction was observed only on transcribed genes (Additional file 1: Fig. S6C). We wondered therefore whether this increase in replication progression could indicate that replication forks would be closely following an ongoing RNAPII and progress faster the faster the RNAPII goes. As such, we would expect also that PCNA and MCM7 ChIP-Seq levels could be directly affected by transcription elongation rates. To assess this, we analyzed PCNA ChIP-Seq clustering genes according to the length of their first intron, as longer first introns correlated with higher transcription elongation rates [57]. For MCM7, we compared the 25% fastest and 25% slowest transcribed genes according to their elongation rates [59]. In both cases, the ChIP-Seq signal was similar in the two groups, suggesting that replication forks progression through genes is not directly related to RNAPII elongation rates (Fig. 5E, Additional file 1: Fig. S6D).

Therefore, we assessed whether the impact of transcription was indirect and correlated with chromatin density. We analyzed histone H3 ChIP-Seq data in our BJ-hTERT cells and HeLa cells [60], analyzing in HeLa separately the ChIP-Seq signals of the variant H3.1, inserted during DNA replication, and the variant H3.3 which is incorporated independently of replication [61]. In BJ-hTERT, we found a progressive reduction in H3 ChIP-Seq levels inside the gene body of genes longer that 100 kb like we observed with PCNA ChIP-Seq signals (Fig. 5F). Analyzing H3.1 and H3.3 in HeLa cells, we found that this progressive reduction was specific of H3.1 and only present at transcribed genes (Additional file 1: Fig. S6E-G). The reduction of histone H3.1 density was not compensated by an increase in the variant H3.3, which is deposited specifically in the gene bodies of actively transcribed genes [62] (Additional file 1: Fig. S6F). We calculated on average a 14.8% reduction for H3.1 along the first 100 kb for genes > 100 kb, similar to the changes measured above for PCNA and MCM7 (Fig. 5B, Additional file 1: Fig. S6B). When analyzing H3.1 signal along fast or slow transcribed long genes, we found that the decrease in H3.1 signal equally was independent of transcription speed, even though transcription elongation rates affected H3.3 levels (Additional file 1: Fig. S5H).

Altogether, this analysis showed that even though the coexistence of transcription and replication can affect genome stability, DNA replication progression could benefit from transcription activity when replicating transcribed genes. As transcription increased its elongation rates along the gene, this reduced chromatin density favoring replication progression. This agreed with previous findings, where gene transcription activation of long genes was also increasing replication speed along these genes [63]. However, although our data would indicate that replication progressed faster along long genes because of the reduced chromatin density, replication forks were also more likely to stall along long genes in proximity of hotspot sites of accumulation of RNAPII (Additional file 1: Fig. S3D).

## Discussion

### Co-existence of RNAPII transcription and DNA replication

RNAPII transcription and DNA replication are two essential for living processes that use DNA as a template. Both processes are active during S-phase, suggesting that an overlap between the two processes is likely to occur, leading to replication stress and genome instability. Previous studies characterizing the nuclear distributions of transcription and replication presented conflicting results about whether transcription and replication occupied the same regions or not [7–9]. In our PLA experiment, we analyzed transcriptionally engaged Ser5-RNAPII and EdU incorporation and found a wide range of PLA foci per cell in Early S phase (Fig. 3A), similar to previously published data [16, 43]. The PLA result could explain the conflicting results from the nuclear distribution analysis by microscopy [7, 8, 16]: whether transcription and replication overlap over a genomic region in S-phase depends on where replication forks are in relation to the genes transcribed in that specific moment in the cell. This is more likely in early S rather than in late S phase because of when replication traverses through transcribed genes. We previously showed that TSSs of transcribed genes could remain occupied by the transcription machinery during replication of the gene, leading to under-replication of TSSs [14]. Here, we showed how transcription also inside genes had a direct impact on replication forks progression. Hotspot sites of RNAPII stalling/pausing appeared dictating where replication forks would stall and pause inside genes (Fig. 2). In parallel, how fast transcription elongated affected replication forks speed both directly and indirectly by altering chromatin density (Figs. 3 and 5). Slowing down transcription elongation in early S phase, when replication traversed a large number of highly transcribed genes, had a direct impact on replication forks speed and progression (Fig. 3C, D). Later during S-phase, when replication was traversing through genes with lower transcription levels, slowing down transcription had much less of an effect (Fig. 3C), also because genes that were replicated were also generally longer [14].

Previous data showed that RNAPII can directly redistribute MCM complexes moving them towards the boundaries of genes and affecting replication origin location [64]. Our data showed that transcription activity also affected the levels of replication factors inside the gene body, linked to changes in replication rates and chromatin density. The impact of transcription on chromatin condensation as well as potential links between transcription and replication rates through chromatin density had already been suggested [65, 66]; hence, our data add to this body of information.

### Impact of transcription-replication collisions on genome instability

We have established an assay to identify genome wide hotspots where replication forks stall and pause, presenting that they showed in general increased genome instability, overlapped with replication fragile sites and with breakage sites of rearrangements found in cancer cells. Importantly, we did not identify specific differences between a replication stalling/pausing hotspot inside a transcribed region and one outside a transcribed region (Fig. 4B), as, in general, stalling/pausing sites were associated with equal levels of γH2AX. If anything, BrdU peaks outside of transcribed regions overlapped more with breakage sites of rearrangements than BrdU peaks inside transcribed regions (Additional file 1: Fig. S5E). However, we cannot exclude a direct impact of transcription on DNA damage repair processes and hence on the frequency of rearrangements, as transcribed regions are preferentially repaired by more accurate and faster DNA damage repair processes than not transcribed regions [67, 68]. Indeed, we showed that inhibition of the DNA damage checkpoint kinases ATM and ATR increased the frequency of rearrangements at BrdU peaks inside transcribed regions.

Our data indicated that both ATM and ATR were important to counteract the formation of rearrangements at transcription-replication collision sites (Fig. 4E). ATM and ATR roles were in part overlapping, as most of the rearrangement events were detected only following treatment with both ATM and ATR inhibitors. Moreover, we observed a slight increase in replication forks stalling at BrdU peaks only following ATM inhibition, more evident at BrdU peaks in transcribed regions (Fig. 4F). This was in agreement with published data that presented increased transcription-dependent genome instability in ATM defective cells and that ATM is activated at sites of transcription-replication conflicts [69, 70]. Altogether, we hypothesized that ATM and ATR might have separate roles in stabilizing replication forks at collision sites and in channeling a broken fork towards more accurate DNA damage repair pathways [71–73]. Previous work showed that ATM and ATR are activated by transcription-replication collisions involving R-loops, with ATM activated by codirectional collisions and ATR by head on ones [55]. As indicated by the analysis of DRIP-Seq data, BrdU peaks did not overall overlap with sites of R-loops accumulation (Additional file 1: Fig. S5A), even though we cannot exclude that R-loops might be formed following the collision [74]. Defining the specific roles of ATM and ATR at transcription-replication collision sites and how they reduce genome instability is an important future avenue of research. It will also be important determining how much transcription-replication collisions contribute to the genome instability present in cancer patients with defective ATM and ATR or whether transcription-replication collisions are responsible for the phenotypes observed in patients with genetic syndromes linked to mutations in ATM and ATR.

## Conclusions

Overall, our data corroborate recent publications evidencing how the coexistence between transcription and replication has crucial consequences for DNA replication itself and for restarting gene transcription following replication [13, 14, 16]. Altogether, our data indicate that the possibility that transcription may impact on replication across a specific gene, is down to the unique combination of transcription levels and transcription elongation rates for that specific gene, and therefore does not happen in all the genes to the same extent. It will also depend on how many RNAPII will be transcribing a specific gene that could be a relatively low number given that the median number of mRNA copies of a gene in a cell is 17 [75]. Nevertheless, even though the likelihood of an encounter between transcription and replication is at any moment low, our genomic and DNA fiber analyses show that this has still a global effect on DNA replication progression (Fig. 3). All in all, the interplay between transcription and replication is complex and their coexistence is not only causing negative consequences for DNA replication, as it is linked to other cellular processes like chromatin compaction and DNA damage repair.

## Methods

Human immortalized BJ-hTERT fibroblasts [76] were cultured in DMEM (Sigma-Aldrich) supplemented with 10% FBS, 2 mM L-glutamine, and penicillin/streptomycin in 5% CO_2_ at 37 °C. Cell identity was authenticated at the origin [76], not tested for mycoplasma contamination.

### Synchronization, DNA labeling, and treatments

Cell growth, synchronization, and labeling of DNA synthesis by BrdU incorporation and sequencing were performed as previously described [14]. At the indicated timepoints, the following drugs were used for the listed time length: DRB (100 μM), α-Amanitin (3 μg/ml), ATMi (10 μM, KU-55933), ATRi (4 μM, AZD6738). BrdU-Seq datasets are available from GEO database, accession number GSE169619.

### Genomic data sets alignment and analysis

Paired-end and single-end reads were aligned to the hg38 genome assembly using Bowtie 2 v.2.3.4.2 on the online platform Galaxy [77]. Fastq files used in this studies were as follows: MCM7 (GSE107248) [58], histone H3.1 and H3.3 (PRJEB27519) [60], BRCA1 (GSE45715) [26], BRCA2 GSE133450 [27], FANCD2 (GSE104464) [28], RAD51 (ENCSR524BUE), XRCC4 (E-MTAB-1241) [24], 53BP1 and Ligase IV (E-MTAB-5817) [25], Orc1 (GSE37583) [34], Orc2 (GSE70165) [35], END-Seq (GSE116321) [19], GLOE-Seq (GSE134224) [18], Oka-Seq (GSE114017) [12], R-loop DRIP-Seq data (GSE57353), and (GSE129907) [51, 52]. For the GLOE-Seq, PCR duplications were removed with the function RmDup on the online platform Galaxy. For the Oka-Seq, mapped bam files were split in the separate strands using the script bam_split.pl [78]. Heatmaps were generated with the function “HeatMap” of EaSeq [79]. In parallel read coverage profiles were generated also using the computational environment EaSeq version 1.101, normalizing the BrdU-Seq file to the Input DNA file with the function “average” [79]. TDF profiles were generated using igvtools on IGV [80] with a window size of 10 bp and window functions of mean, and igvtools “combine data tracks” was used also to generate difference profiles like in the case of peaks affected by DRB or α-Amanitin treatment.

Total RNAPII (Rpb1 NTD antibody, Cell Signaling) and PCNA (PC10, The Francis Crick Institute) ChIP-Seq were performed in synchronized cells as in [14]. γH2AX, H2AX, and H3 ChIP-Seq was performed as in [14]. γH2AX and histone H2AX levels were quantified over all genes using the function “Quantify” of EaSeq [79]. γH2AX levels were normalized to H2AX levels in each repeat, before being averaged from 2 independent experiments and plotted. To correlate read counts of the BrdU-Seq with the read counts of the Chr-RNA-Seq or γH2AX ChIP-Seq, we used the function “Correlate” of EaSeq [79] with a window size of 1 kb, including windows with at least 10 reads, for each indicated timepoint.

### Peak calling

Peaks were called in BrdU samples against the Input DNA, and in the γH2AX samples against histone H3 ChIP-Seq, using MACS2 v.2.1.0 [81] with human genome size and the following parameters: -m 8 30, -p 0.00001. Around 18,000 peaks were called in early S: 9000 in early/mid S, 3000 in mid S, 1500 in late S, and 2700 in late S/G2. The -intersectBed command of Bedtools software [82] was used to get the overlaps for the transcribed genes and peaks in each replication time point. In the early S phase timepoint, this resulted in 2428 peaks in codirectional genes, 2452 peaks in head on genes, and 2091 peaks in undetermined genes. To shuffle BrdU peaks randomly within the same genes in which they have been identified, we used the function “bedtools ShuffleBed” from Galaxy, limiting the Genome file only to the coordinates of the genes containing a BrdU peak.

### mRNA-Seq

Cells were synchronized by serum starvation as described before [14] and treated at the indicated timepoints with DMSO, DRB (100 μM), or α-Amanitin (3 μg/ml) for 1 h, collected as above with RNA extracted with RNeasy MiniKit (QIAGEN). RNA libraries were prepared according to NEBNext Ultra II Directional RNA Library Prep Kit (NEB) instruction and sequenced on Illumina NextSeq system in a single lane on a single-end read run. Quality control analysis on raw sequence data derived from BrdU-Seq and mRNA-seq was performed using fastqc [77]. Single-end RNA-seq data were aligned to the hg38 genome assembly using STAR v.020201 [83] with options “--alignIntronMax 500000,” “--outFilterScoreMinOverLread 0.3,” and “--outFilterMatchNminOverLread 0.3.” Reads were trimmed to remove RNA contamination via BBDuk [84]. BAM files were sorted and indexed using SAMtools v.1.4 [85]. Counts for each transcribed gene were computed by featureCounts [86] using the annotation of the GENCODE genes (GRCh38.p10). Differential expression analysis in RNA-seq data was conducted by using the package DESeq2 [87]. This tool provides methods to test for differential expression by use of negative binomial generalized linear models; the estimates of dispersion and logarithmic fold changes incorporate data-driven prior distributions. Genes were defined as differentially expressed following DRB or a-Amanitin treatment, if they were > 2-fold change (FC) or < 2 FC compared to the DMSO treated sample, with *p*-value < 0.05 and *q*-value < 0.05. The RNA-Seq data for the DMSO, DRB, and α-Am treatments are available from GSE169596.

Assessment of the targets downstream of the p53 pathways was done using the expression levels calculated from the mRNA-Seq. For the analysis of the expression levels of histone genes, the same RNA used for the mRNA-Seq has been reverse transcribed with the qSCRIPT cDNA synthesis Kit (Quantabio), followed by RT-PCR as above, with primers at specific histone genes and normalized to the expression levels of RPLP0 as indicated in the Additional file 4: Table S3.

### Proximity ligation assay

Cells were seeded on 9 mm coverslips and were pulsed with 125 μM EdU for 10 min at the indicated timepoint. Fixation and permeabilization were performed as described [43]. After permeabilization, cells were washed in PBS twice for 5 min each. The “Click-chemistry” reaction was performed using Click-iT RNA Alexa Fluor 594 Imaging Kit following the manufacturer’s instructions with slight modification (Thermo Fisher Scientific). When preparing Click-iT reaction cocktail, Alexa Fluor azide was replaced with either DMSO (no click control) or 10 μM of Biotin Azide (Stratech). Click reaction was performed in a humid chamber at room temperature for 30 min. After the click reaction, cells were washed in 10 cm dishes with PBS for 5 min. Cells were blocked with 10% FBS/PBS for 1 h at room temperature and then incubated with primary antibody in 10% FBS/PBS overnight at 4 °C (4H8, 1:250; Rabbit anti-Biotin antibody, 1:500). Cells were again washed in 10 ml wash buffer A solution twice for 5 min each with gentle agitation and incubated in a pre-mixed solution of PLA probe anti-mouse minus and PLA probe anti-rabbit plus (Sigma-Aldrich) in a pre-heated humidity chamber for 1 h at 37 °C. The Duolink In Situ Detection Reagents (Red) were then used to perform the PLA reaction according to manufacturer’s protocol. Slides were mounted in Duolink In Situ Mounting Medium with DAPI and imaged on a Nikon Eclipse E600 Fluorescence Microscope using a Nikon Plan Apo × 60 (1.3 numerical aperture) oil lens, a Hamamatsu digital camera (C4742-95), and the Volocity acquisition software (Perkin Elmer). The number of PLA foci was counted.

### Transcription elongation rates calculations

Transcription elongation rates were calculated similar to [5]. BJ-hTERT cells were seeded in 60-mm dishes, one dish per sample. Cells were treated for 3.5 h with 100 μM DRB and then washed twice in PBS before being incubated with fresh complete medium for transcription to restart. Twenty minutes after restart, cells were either left in complete medium with DMSO or incubated with 100 μM DRB or 3 μg/ml α-Amanitin. Every 10 min, a cell dish was lysed directly in RLT buffer + β-Mercaptoethanol and collected on ice with a scraper. RNA was extracted with RNeasy MiniKit (QIAGEN), according to manufacturer’s recommendations. One microgram of RNA was reverse transcribed with random hexamers with the SuperScript III Reverse Transcriptase kit (Invitrogen). Primers were designed at specific distances from the TSS (1 kb, 15 kb, 40 kb, 60 kb, 80 kb) and normalized to the expression levels of RPLP0 as listed in the Additional file 4: Table S3. Pre-mRNA levels were assessed by quantitative RT-PCR using SensiFast SYBR Lo-ROX kit (Bioline) and CFX96 Real-Time System (Biorad). When transcription levels were back to the control untreated ones, it meant that transcription progression had reached that specific position along the gene. The distance traversed between one primer set and the following one was divided by the time needed to reach the further away primer set, giving the elongation rate over the specific section of the gene. Elongation rates were averaged over different parts of the gene in an average elongation rate for the gene in control cells and cells treated with the transcription inhibitors. Results shown are average means of three independent replicates, ± standard error of the mean.

### DNA fiber assay

Cells were synchronized by serum starvation as above and treated with DMSO, 100 μM DRB, and 3 μg/ml α-Amanitin for 1 h, at the indicated timepoints. Cells were then pulsed with 25 μM CldU (Sigma-Aldrich) and 250 μM IdU (Sigma-Aldrich) for 20 min each in the presence of indicated drugs (DMSO/DRB/α-Amanitin). Cells were washed twice with ice-cold PBS and harvested by trypsinization and resuspended in 0.5 ml of ice-cold PBS. Two microliters of cells (5 × 10^5^ cells/ml in PBS) were put onto microscope slides until edges of the drop became crinkly. Cell lysis was prepared by adding 7 μl of spreading buffer (0.5% SDS, 200 mM Tris–HCl pH 7.4 and 50 mM EDTA) on top of cells and mixing. After incubating cell lysis for 2 min at room temperature, DNA fiber spreads were prepared by tilting the slides to allow the cell lysate to move until the end of the slides. DNA fiber spreads were left at room temperature for at least 2 min to dry followed by fixation in methanol/acetic acid (3:1) for 10 min. The slides were air-dried for 10 min and stored at 4 °C until use. Slides were washed twice with water and once with 1 ml freshly made 2.5 M HCl followed by denaturation with 2.5 M HCl for 75 min. HCl-treated fiber spreads were incubated with rat anti-bromodeoxyuridine (detects CldU, Abcam, 1:500) and mouse anti-bromodeoxyuridine (detects IdU, BD BioScience, 1:500) at room temperature for 1 h, fixed with 4% paraformaldehyde (PFA) for 10 min at room temperature. The DNA fibers were then incubated with anti-rat IgG AlexaFluor 555 (Thermo Fisher Scientific, 1:500) and anti-mouse IgG AlexaFluor 488 (Thermo Fisher Scientific, 1:500) for 1.5 h. Images were acquired as described above. Images were merged using GIMP and analyzed using ImageJ. In each independent experiment, > 130 fibers were measured per condition in each replicate.

### Overlap with CFS and ERFS

The file containing the locations of CFS in hg19 was downloaded from [53]. The file with the ERFS location was in mouse mm9 genome, downloaded from [54]. Both lists were converted to hg38 using the UCSC built-in liftover function. Both lists were then used to overlap with the 5 timepoints BrdU-Seq peaks in S-phase. To determine whether the overlap was significant and not the result of random coincidence, pybedtools [88] was used to shuffle bed files with 10,000 iterations, and then overlap with the CFS and ERFS lists was analyzed. The normal distribution of the randomly overlapped regions was produced with different statistics calculated including the empirical *p*-value, which was used to determine the significance of overlap between the specific replication fork stalling regions and the fragile sites.

### Distance analysis and hexamer frequency

To calculate the distance between BrdU peaks and specific features like origins of replication or exons, we used the function “bedtools ClosestBed” on Galaxy. The list of BrdU peaks was compared against the list of the core and stochastic origins from [36], the list of Ini-seq identified origins from [29], and the list of replication zones identified by Repli-Seq [89] as well as the list of UCSC annotated exons in hg38. For the analysis of the frequency of hexamers, we used the function “Hexamer frequency” on Galaxy. For the list of the peaks affected by DRB and α-Amanitin, we overlapped the list of genes affected in the mRNA-Seq analysis with the list of BrdU peaks called in the first timepoint using the ClosestBed function as previously mentioned. Identical approach was used for peaks affected by ATRi, using the list of genes affected by ATRi from [56].

### Breakpoint analysis

For the rearrangement breakpoint analysis, we downloaded the Structural Genomic Rearrangements datafile (CosmicStructExport.tsv, release version v92) from the Cosmic website (https://cancer.sanger.ac.uk/cosmic). Additional data manipulation was conducted, using an in-house script in R, over breakpoints regions in order to create two appropriated windows (left and right) for downstream analysis. To associate the nearest feature between the breakpoints and peaks, based upon genomic distance measures, the *closest-features* function from BEDOPS v2.4.39 [90] was used. In order to find the overlapping between the two inputs (breakpoints and peak), the option “*--dist* “ was implemented considering any distance of 0 as an overlap 1.

For the overlap between BrdU peaks and breakpoint analysis, all the statistical tests were performed in R (4.0.3). Given samples $A$ and $B$, the null and alternative hypotheses are respectively ${H}_{0}:\overline{{d}_{A}}=\overline{{d}_{B}}$ and ${H}_{1}:\overline{{d}_{A}}\ne \overline{{d}_{B}}$, where $\overline{{d}_{X}}$ denotes the sample mean of the Boolean vector $[{{\varvec{d}}}_{X}=0]$, while ${{\varvec{d}}}_{X}$ is the BrdU peaks/breakpoint in sample $X$. Consequently, the statistics is defined as ${t}^{*}= \overline{{d}_{A}}-\overline{{d}_{B}}$. To relax the distributional assumptions, statistical tests were performed by employing the bootstrap permutation procedure with at least 10,000 times of resampling for each test. Once *p*-values are computed, Benjamini–Yekutieli (BY) correction was applied in order to mitigate the type I error and consider any potential correlation across the multiple tests [91]. The in-house script is available via GitHub, https://github.com/rojasp/Positive-and-negative-impact-of-RNA-Pol-II-transcription-on-DNA-replication-progression. No other scripts and software were used other than those mentioned in this “Methods” section.

### Rearrangement PCR

Cells were treated 14 h after serum starvation release when in early S-phase for 2 h with DMSO, ATMi, and/or ATRi or for 1 h with DRB or α-Amanitin. After drug treatments, cells were washed in PBS, released into complete medium, and harvested 6 h later by trypsinization; by that point, cells will be towards the end of their S-phase. DNA was extracted with PureLink Genomic DNA Mini Kit (Invitrogen) following manufacturer’s protocol. Frequency of rearrangements was assessed by RT-PCR as above described comparing the ATMi and/or ATRi treated sample to the DMSO control treated sample. As for each BrdU peak, we could often identify several specific rearrangements, one forward primer was designed upstream of the lower coordinate breakage site, and two separate reverse primers downstream of the higher coordinate breakage site. The two separate reverse primers were designed strand specifically to identify either deletions, inversions or translocations, available in the Additional file 4: Table S3.

### Quantification and statistical analyses

The number of experimental repeats $\ge$ 3 is indicated in the figure legends. For the NGS files, it was two biological repeats of the full timepoint sets of BrdU-Seq each for treatment with ATMi, ATRi, DMSO, DRB, and α-Amanitin, one for the ChIP-Seq of RNAPII and PCNA. The repeats were all assessed for correlation before being analyzed together and averaged were specified. Student *t*-test and Mann–Whitney *t*-test were calculated using the software Prism (GraphPad).

### Supplementary Information

Additional file 1: Fig. S1. Identification of replication fork stalling/pausing hotspot sites. Fig. S2. Overlap of BrdU peaks with other genomic features. Fig. S3. Transcription-replication collision sites. Fig. S4. Transcription elongation rates directly affected replication progression. Fig. S5. BrdU peaks were hotspots of genome instability sites. Fig. S6. Transcription activity reduced chromatin density increasing DNA replication rates.

Additional file 2: Table S1. Genes differentially expressed following DRB or a-Am treatment.

Additional file 3: Table S2. Statistical analysis of BrdU peaks overlap with breakpoints.

Additional file 4: Table S3. List of primers used in this paper.

Additional file 5. Review history.

## Data Availability

The accession number for all the genomic data files reported in this paper is GSE169620 [92]. The RNA-Seq data for the DMSO, DRB, and α-Am treatments are available from GSE169596 [93]; the BrdU-Seq data of DMSO, ATMi, and ATRi-treated samples are available from GSE169619 [94]; and the ChIP-Seq data of RNAPII and PCNA are available from GSE267038 [95]. The in-house script is available via GitHub [96] and from Zenodo [97].
