# Supplementary material for: Genome-wide identification of replication fork stalling/pausing sites and the interplay between RNA Pol II transcription and DNA replication progression
Source: Genome Biol. 2024 May 21;25:126. doi: 10.1186/s13059-024-03278-8 (PMC11106976; doi:10.1186/s13059-024-03278-8)
Supplement: Supplementary file 1 — Additional file 1: Fig. S1. Identification of replication fork stalling/pausing hotspot sites. Fig. S2. Overlap of BrdU peaks with other genomic features. Fig. S3. Transcription-replication collision sites. Fig. S4. Transcription elongation rates directly affected replication progression. Fig. S5. BrdU peaks were hotspots of genome instability sites. Fig. S6. Transcription activity reduced chromatin density increasing DNA replication rates. [file 13059_2024_3278_MOESM1_ESM.docx]

**Figure S1. Identification of replication fork stalling/pausing hotspot sites**

1. Average metagene profile for BrdU-Seq levels at BrdU peaks called in the indicated timepoints, at the timepoint they are identified and all the following ones like in Fig 1C. (B) Average metagene profile for BrdU-Seq in all the timepoints and γH2AX ChIP-Seq at BrdU-Seq peaks and the γH2AX ChIP-Seq peaks. (C) Average metagene profiles and heatmaps for ChIP-Seqs of 53BP1, Ligase IV and XRCC4 (*Aymard et al., 2014*; *Clouaire et al., 2018*), BRCA1 (*Gardini et al., 2014*), BRCA2 (*Gruber et al., 2019*), FANCD2 (*Okamoto et al., 2018*) and RAD51 (Encode) at BrdU peaks +/- 2.5 kb. (D) Average metagene profiles and heatmaps for GLOE-Seq from *Sriramachandran et al*. at BrdU peaks called in each timepoint. (E) Average metagene profiles for ChIP-Seqs of Orc1 and Orc2 (*Dellino et al., 2013*; *Miotto et al., 2016*) at TSS of transcribed genes > 100 kb and BrdU peaks +/- 10 kb. (F) Heatmaps for Oka-Seq from *Chen et al.* 2019 at BrdU peaks called in each timepoint. (G) Average metagene profiles and heatmaps for ChIP-Seqs of BRCA1 (*Gardini et al., 2014*), BRCA2 (*Gruber et al., 2019*), FANCD2 (*Okamoto et al., 2018*) and RAD51 (Encode) at TSS of genes > 100 kb +/- 2.5 kb. (H) Frequency of overlap and distances calculated between BrdU peaks identified in each timepoint and the closest core and stochastic origins from *Akerman et al*., Ini-Seq origins from *Langley et al*. and Oka-Seq from *Wu et al*. (I) Average metagene profile for PCNA ChIP-Seq at BrdU peaks overlapping with origins identified in H. (L) Ratio of Watson and Crick strands at BrdU peaks overlapping with origins identified in H (right), with genes > 100kb shown as positive control, showing that the ratio also across BrdU peaks overlapping with origins identified in H is on average 1, meaning that on average they are passively replicated.

**Figure S2. Overlap of BrdU peaks with other genomic features**

(A) Frequency of hexamers present across BrdU peaks, with highlighted the four most frequent ones. (B) Heatmap analysis for BrdU-Seq and histone H3 across all BrdU peaks. (C) Heatmap and average metagene profile for ChIP-Seq signal for the listed histone modifications and CTCF at BrdU peaks called in all timepoints +/- 2.5kb, sorted by BrdU-Seq signal intensity; in parallel the same histone modifications and CTCF are shown at positive control sites like the TSS +/- 10kb of genes replicated in Early S or of all genes in the genome for H3K27me3.

**Figure S3. Transcription-replication collision sites**

(A) Average metagene and heatmap profiles for Chr-RNA-Seq at BrdU peaks +/- 2.5 kb in transcribed regions according to the strand that the gene is transcribed, ‘+’ or ‘-‘. (B) Heatmap profiles for Chr-RNA-Seq at BrdU peaks +/- 2.5 kb at BrdU peaks randomly shuffled inside the same genes where genuine BrdU peaks were identified. (C) Correlation analysis performed with EaSeq between the number of reads for Chr-RNA-Seq and BrdU-Seq at each timepoint in a 1k window, and for γH2AX ChIP-Seq and BrdU-Seq at each timepoint as a positive control. (D) Average heatmaps and metagene profiles for ChIP-Seq of total RNAPII in the G1/S and Early S timepoints across BrdU peaks identified in the first timepoint, separated as codirectional, head on or undetermined (Undet). (E) Distance calculated for BrdU peaks in each timepoint to the closest annotated exon. (F) Gene lengths for all genes replicated in the Early S timepoint towards genes replicated in the Early S timepoint with a BrdU peak. (G) Transcription levels in fragments per kb per million (FPKM) according to when transcribed genes are replicated and frequency of when transcribed genes are replicated. Mann-Whitney t-test; **** => p-value < 0.0001.

**Figure S4. Transcription elongation rates directly affect replication progression**

(A) Schematic of primer positions and experimental design to measure elongation rates along SPTAN1 following release from DRB wash out in DMSO, DRB (100 μM) or α-amanitin (α-Am, 3 μg/ml) 20 minutes after transcription restart; n= 3, average mean +/- SEM. (B) IdU/CldU ratio for fibers measured in Fig 2C; box whiskers plots with line at the median 2.5-97.5 percentile, Mann-Whitney t-test. (C) Summarizing table with the number of significantly differentially up- or down-regulated transcripts in Early S and Mid S following treatment for 1 hour with DRB (100 μM) or α-amanitin (α-Am, 3 μg/ml), and venn diagram of the differentially up- or down-regulated transcripts in Early S and Mid S following treatment for 1 hour with DRB (100 μM) or α-amanitin (α-Am, 3 μg/ml), highlighting the large overlap between genes by DRB affected at both timepoints, and that only two genes are affected by both drugs in both timepoints. (D) Quantification as fold change (FC) compared to CTR DMSO from the mRNA-Seq of the listed p53 target genes following treatment in Early S and Mid S for 1 hour with DRB (100 μM) or α-amanitin (α-Am, 3 μg/ml); average mean, Student t-test. (E) Quantification of the expression levels of the listed histone genes compared to the housekeeping gene RPLP0 following treatment in Early S and Mid S for 1 hour with DRB (100 μM) or α-amanitin (α-Am, 3 μg/ml); average mean +/- SD, Student t-test. (F) Quantification of transcription activity by Chr-RNA-Seq from *Wang et al.*, across genes replicated in Early S clustered according to their gene length as fragments per kilobase per million (FPKM). (G) Quantifications of the BrdU-Seq signals from TSS to TTS in the DRB or α-amanitin samples as fold changes compared to the DMSO one for genes > 100kb that are replicated in the first timepoint but are not transcribed (417 genes); paired nonparametric t-test. (H) Average metagene profiles of BrdU-Seq levels in Early S following treatment for 1 hour with DRB (100 μM) or α-amanitin (α-Am, 3 μg/ml) while cells are pulsed with BrdU, at the BrdU peaks called in Early S +/- 1 kb. * => p-value < 0.05 ** => p-value < 0.01, *** => p-value < 0.001, **** => p-value < 0.0001.

**Figure S5. BrdU peaks are hotspots for genome instability sites**

(A) Heatmap analysis and average metagene profile for R-loop ChIP-Seq (DRIP-Seq) signal at BrdU peaks +/- 1kb called in the first timepoint separated by peak type, in fibroblasts (*Lim et al., 2015*) and in U2OS cells (*Wu et al., 2020*). (B) Enrichment analysis of occurrence of BrdU peaks called in all the timepoints with common fragile sites (CFS) and early replicating fragile sites (ERFS), compared to a randomized overlap generated by 10000 iterations of randomly arranged BrdU peaks. (C) Replication timing for genes listed in CFS and ERFS regions according to our previous BrdU-Seq and Chr-RNA-Seq analysis from *Wang et al*. (D) Comparison of the frequency of increased overlap of BrdU peaks called in all timepoints with breakpoints, comparing BrdU peaks in not transcribed regions towards BrdU peaks in transcribed region, measured in fold change with relative p-value. (E) Comparison of the frequency of increased overlap of BrdU peaks called in all timepoints with breakpoints, comparing BrdU peaks in transcribed regions towards other types of BrdU peaks in transcribed region, measured in fold change with relative p-value, analyzing separately the left (L) and the right (R) breakpoint for each rearrangement; highlighted in red are the only comparisons highlighted that are not statistically significantly different. (F) IGV snapshots at BrdU peaks over genes that are up- or down-regulated following treatment with ATR inhibitor, showing how changes to the expression of these genes changes also the levels of BrdU-Seq signal across these peaks. (G) As for Fig 4E but treating cells in Early S for 1 hour with DRB (100 μM) or α-amanitin (α-Am, 3 μg/ml); n$\geq3$, average mean +/- SEM, Student t-test. * => p-value < 0.05 ** => p-value < 0.01.

**Figure S6. Transcription activity reduces chromatin density increasing DNA replication rates**

(A) Average metagene profile for histone MCM7 ChIP-Seq from *Sugimoto et al*., at TSS +/- 100kb (left panel) and TSS +/- 10kb (right panel) of transcribed genes clustered by gene length. (B) Schematic on how the increase in replication rates across the gene length has been calculated. (C) Average metagene profile for histone MCM7 ChIP-Seq from *Sugimoto et al*., at TSS +/- 100kb (right panel) and from TSS to TTS (left panel) for genes > 100kb transcribed or not transcribed. (D) As for C) for genes > 100kb transcribed separated as fast or slow transcribed according to *Fuchs et al*. (E) Average metagene profile for histone H3.3 and H3.1 ChIP-Seq from *Clement et al*., at TSS +/- 100kb for transcribed genes > 100kb. (F) Average metagene profile for histone H3.1 and histone H3.3 ChIP-Seq from *Clement et al*., at TSS +/- 100kb 100kb of transcribed genes clustered by gene length. (G) As for C) with ChIP-Seq of histone H3.1. (H) Average metagene profile for histone H3.1 and H3.3 ChIP-Seq from *Clement et al*., at TSS +/- 100kb of transcribed genes > 100kb separated by transcription elongation rates in fast or slow according to *Fuchs et al*.
