# Supplementary material for: Genome-wide identification of replication fork stalling/pausing sites and the interplay between RNA Pol II transcription and DNA replication progression
Source: Genome Biol. 2024 May 21;25:126. doi: 10.1186/s13059-024-03278-8 (PMC11106976; doi:10.1186/s13059-024-03278-8)
Supplement: Supplementary file 4 — Additional file 4: Table S3. List of primers used in this paper. [file 13059_2024_3278_MOESM4_ESM.docx]

| **Primer list:** |  |
| --- | --- |
| Sptan1 1kb For | GTGCTTGCATGGTATGTGGC |
| Sptan1 1kb Rev | CGACTCGAAAGACCCAGTCC |
| Sptan1 15kb For | CCACCGCTTCAAGGAACTCT |
| Sptan1 15kb Rev | TTTGGCAGGAATCCCAGGAG |
| Sptan1 40kb For | TGTGCAGGACACGCATAACT |
| Sptan1 40kb Rev | GACCTCTTCCCTTGTGAGCC |
| Sptan1 60kb For | CCTGCTGGCATCCGAAGATT |
| Sptan1 60kb Rev | CCTGAGGCCACAACCTTTGA |
| Sptan1 80kb For | TATGGTGGAAGAGTCGGGGA |
| Sptan1 80kb Rev | GGCCAAGGGAAAAGGGTACA |
| Cod1 F | CCATGTGAGGCTTATGCTCT |
| Cod1 R1 | TAGGGAAATCATGGGGTCTC |
| Cod1 R2 | AAAGGATAGGTGGGCTGTGC |
| Cod2 F | GCCAGTCTGAAAAGGATACA |
| Cod2 R1 | GATATATCTCTCCCTTACCC |
| Cod2 R2 | GCGTCATGACCATAATCATC |
| Cod3 F | AGGCAGAAGGCTTTTGGAAG |
| Cod3 R1 | TTGGCACTCCTGGTTTCCAG |
| Cod3 R2 | GACAAGGCAGTTATATTAGG |
| Cod4 F | TACACAGAGGACAAGGGAAC |
| Cod4 R1 | CTGTCAATATCTACACTGGG |
| Cod4 R2 | GACAAAGGGAATAGTGAAAATG |
| Cod5 F | CTGTCATCTCCATCATCATC |
| Cod5 R | ACAGGGACAATGGTGGTAG |
| H1 F | CATGTGCAGCCTGATATGTG |
| H1 R1 | TCTCCTACTATGATTCTGGG |
| H1 R2 | TAGACAACCCACAATCATAG |
| H2 F | TGCTATGCAATACTACG |
| H2 R1 | CTGTTCCTTCTATGCTACC |
| H2 R2 | GCCTGATACTGTATAGAATG |
| H3 F | CTGGTGAATCAGAGTATATG |
| H3 R | GCATATACTCTATCACTGC |
| H4 F | GCTCTGATCACATCATCACC |
| H4 R1 | GATGATGGTGATGGTGACAG |
| H4 R2 | TTCATCACCACCATCATCCC |
| H5 F | ATCATAGTGTCCCAGTGTGC |
| H5 R1 | ACTTTGGGTTTGTAGTCTCC |
| H5 R2 | ATCATAGTGTCGCAGTGTGC |
| HIST1H2BD Total For | GTGGATCCCACCCAAATCCA |
| HIST1H2BD Total Rev | TCCTTCCCCTCGGTAACCTT |
| HIST1H2AG For | ACAAGGCGAAGGGCAAGTAA |
| HIST1H2AG Rev | AATCTGCGGTGTTCGCTACA |
| HIST1H1B For | GAGCGCAATGGCCTTTCTTT |
| HIST1H1B Rev | CCTTCTTGGCTTTGGGCTTG |
| HIST4H4 For | ACCCTTTGATGTCGCCTTCC |
| HIST4H4 Rev | CCCCCTCCTTGAGGTCTAGT |
| HIST3H2A For | TATCTCGAGAAAAGAGCCGCA |
| HIST3H2A Rev | GCAAGTATGCAGCCCAATCA |
| RPLP0 F1 | AGCCCAGAACACTGGTCTC |
| RPLP0 R1 | ACTCAGGATTTCAATGGTGCC |
