# Supplementary material for: Genome-wide identification of replication fork stalling/pausing sites and the interplay between RNA Pol II transcription and DNA replication progression
Source: Genome Biol. 2024 May 21;25:126. doi: 10.1186/s13059-024-03278-8 (PMC11106976; doi:10.1186/s13059-024-03278-8)
Supplement: Supplementary file 5 — Additional file 5. Review history. [file 13059_2024_3278_MOESM5_ESM.docx]

Review History

**First round of review**

**Reviewer 1**

**Were you able to assess all statistics in the manuscript, including the appropriateness of statistical tests used?**

No, I do not feel adequately qualified to assess the statistics.

**Comments to author:**

In this manuscript the authors aim to get more insight into how RNAPII transcription affects DNA replication progression and its impact on genome instability. They used the BrdU-seq and Chr-RNA-seq datasets from their previous work (Wang et al., 2021 Cell Rep 34: 108759) to identify sites of accumulated BrdU signal and RNA reads across several S-phase fractions and showed their overlap with previously identified fragile sites and with breakpoints of genomic rearrangements found in cancers. I found the questions raised by the authors really interesting, but regret not to be as supportive regarding the main conclusions of their work.

My major concern focuses on the nature of what they define as replication fork stalling/pausing sites. If we considered that DNA replication proceeds at an average time of 20 nt/sec, 1h BrdU pulse will label approximately 72 kb of DNA, allowing the mapping of replication timing profiles and the detection of 1.5 kb gaps of unreplicated DNA at TSSs of hundreds of genes, as they did in their previous work. When the authors now performed a peak calling on those samples, they identify prominent, narrow peaks of accumulated BrdU reads approximately 1 kb length, specially at early-S, which they interpreted as fork stalling/pausing sites (Fig 1C and 1D). To detect such defined events on the top of a 72 kb BrdU-labeled region, they must occur on most cells at the same genomic location and persist for nearly 2h to be still detectable on the next time point. However, in the browser examples shown in Fig 2C, BrdU peaks appear discontinuous over 6-7 kb regions, raising the question of what they truly represent. Giving the similarly unexpected shape of the Chr-RNA reads on the same regions (discontinuous, not preferentially accumulated at exons as usually happens due to co-transcriptional splicing) and their physical proximity (Fig 2), I wonder whether, instead of replication fork pausing/stalling events as the authors claim, BrdU peaks might account for initiation events distinct from canonical replication initiation sites (that, at transcribed regions might be RNA-primed). One way to test this will be by analyzing the location of BrdU peaks relative to high-resolution Repli-seq regions (like in Zhao et al 2020 Genome Biol 21: 76). Another possibility fitting with the rest of the observations shown in the manuscript is that BrdU peaks represent preferential sites of repair synthesis after DNA damage, independent of transcription. The authors should consider and test other possibilities for interpreting their data.

As mentioned above, I also do not understand what the short RNA reads from the Chr-RNA-seq experiments represent since they do not cover the whole gene body nor accumulate at exons like chromatin-bound RNA reads do (Fig 2). Could the authors show the TSS-centered read coverage of the corresponding genes from panels 2B and 2E?

Other points:

1. The message of the paper is built around the impact of RNAPII transcription on DNA replication, however only 38% of the BrdU peaks are in genes. Since their association with fragile sites and breakpoints is indistinguishable regardless they are genic or not, and considering other potential explanations for their origin, I think the authors should consider changing the focus of the text (and title).
2. Regarding the above, the authors correlate the BrdU peaks with multiple published datasets (Fig 1F, 1G, S1, S2). In all cases, the cell lines from which the data were derived should be clarified in the text. This is especially relevant in the case of CFS, as they are cell type specific (Michelle Debatisse´s work).
3. Relevant controls to support their claim that BrdU peaks are indeed hotspots of DNA damage would be showing BrdU-seq, P-H2AX-seq and GLOE-seq signals at TSS in Fig 1 BRCA1, BRCA2, FANCD2 and RAD51 in Fig S1.
4. Why did the authors use S5P-RNAPII instead of S2P-RNAPII for the PLA experiments in Fig 3A? Wouldn´t the latter be more informative of the co-existence of on-going replication and transcription?
5. In Fig 3C the maximum BrdU signal occurs at the TSS of genes in cells treated with transcription inhibitors. DMSO unnormalized signal should be also shown in these metagenes (see also my comment in point 3) to better understand what we are dealing with here.
6. In lines 549-551 the authors conclude that "changes in RNAPII elongation directly affect DNA replication progression and/or efficiency, with a direct correlation to genome instability levels in genes". To claim a direct correlation, they should perform a pair-wise comparison between BrdU-seq reads and P-H2AX reads at the same genes.
7. The analyses of PCNA coverage along genes of different lengths is intriguing. How to reconcile the clear decrease in PCNA signal along genes >100 kb indicative of faster moving forks with an increase in BrdU signal (Fig 3A-B)?
8. Fig 5E; how the analysis is performed needs clarification in the text and methods.

**Reviewer 2**

**Were you able to assess all statistics in the manuscript, including the appropriateness of statistical tests used?**

Yes, and I have assessed the statistics in my report.

**Comments to author:**

Rojas et al present work addressing important issues about replication and transcription machines cohabiting in genic regions. They use whole genome approaches analysing nascent replication and transcription products as well as ChIP to correlate replication, transcription and DNA damage, mainly. Their conclusions are that ongoing transcription and replication co-exist. Pausing of the two machineries coincide. Active transcription may accelerate replication due to lowering nucleosome density. Pausing sites correlate with several signs of DNA damage and genome re-arrangements. The prevention of re-arrangements requires DNA damage signalling.

The argumentation line of the manuscript is hard to follow. The manuscript is complicated and lengthy and the language is imprecise. Whilst the first two figures (including 3a) seem relatively clear, with sufficiently strong experimental effects measured, the manuscript becomes increasingly unclear from this point. Partly, effects measured seem minute, for example RNAi experiments in Fig 3. Moreover, similar experiments seem to be displayed in different ways, which makes it even more difficult to follow. Furthermore, it remains often unclear to me what the authors intend to state with specific experiments. Conclusions are not clearly formulated.

Sometimes it remains unclear to me what the power of particular experimental techniques are, particularly in light of weak effects measured. I appreciate that averaging effects over the whole genome (or all genes) dilutes effects on individual genes, but lends strength to the experiments by their statistical power. However, this finds its limits when effect size starts to affect the credibility of conclusions. A mixture of two measures could lend strength on the experimental results:
1) providing data on the power of experiments by creating clearly defined predictable situations. For example, aphidicolin slows replication predictably and tunably. DNA damage can be induced to test the effect on transcription and replication.
2) complementing whole genome analyses with analyses of specific genes. This could lead to stronger effects. Ideally, the authors observe tunable genes (endogenous or a tet-inducible transgene, for example) that they could switch on and off and analyse the consequences. Manipulation is tried in figure 2, which is appreciated. But the effects were small (RNAi) and not gene-specific but systemic.

I apologise that I cannot support publication of the manuscript in the current form.

**Authors Response**

**Point-by-point responses to the reviewers’ comments:**

Reviewer #1:
In this manuscript the authors aim to get more insight into how RNAPII transcription affects DNA replication progression and its impact on genome instability. They used the BrdU-seq and Chr-RNA-seq datasets from their previous work (Wang et al., 2021 Cell Rep 34: 108759) to identify sites of accumulated BrdU signal and RNA reads across several S-phase fractions and showed their overlap with previously identified fragile sites and with breakpoints of genomic rearrangements found in cancers. I found the questions raised by the authors really interesting, but regret not to be as supportive regarding the main conclusions of their work.

*Reply: We appreciate that the reviewer considered the questions raised interesting, and we hope that the additional data and analyses provided can support the main conclusion of our work.*

My major concern focuses on the nature of what they define as replication fork stalling/pausing sites. If we considered that DNA replication proceeds at an average time of 20 nt/sec, 1h BrdU pulse will label approximately 72 kb of DNA, allowing the mapping of replication timing profiles and the detection of 1.5 kb gaps of unreplicated DNA at TSSs of hundreds of genes, as they did in their previous work. When the authors now performed a peak calling on those samples, they identify prominent, narrow peaks of accumulated BrdU reads approximately 1 kb length, specially at early-S, which they interpreted as fork stalling/pausing sites (Fig 1C and 1D). To detect such defined events on the top of a 72 kb BrdU-labeled region, they must occur on most cells at the same genomic location and persist for nearly 2h to be still detectable on the next time point.

*Reply: The reviewer is right about the fact that BrdU peaks need to be frequent events to be detected above the average BrdU-Seq signal. The fact that there is also clear signal for gH2AX at these sites indicates that these locations are conserved and frequent across a population of cells. We have tried to estimate how many cells would actually need to have a replication fork stalling in these sites to give rise to such an increase in BrdU signal. However, we could not find a suitable tool to perform such an analysis. Hence, we know that they are frequent, hence we are clearly picking up hotspots where replication forks stall/pause, but we can’t estimate exactly how frequent they are.*

However, in the browser examples shown in Fig 2C, BrdU peaks appear discontinuous over 6-7 kb regions, raising the question of what they truly represent.

*Reply: In panel 2C we are showing multiple examples of BrdU peaks, but for example in the third panel we are showing a region where three separate BrdU peaks have been identified (indicated by the light blue bar over the BrdU-Seq profile).*

Giving the similarly unexpected shape of the Chr-RNA reads on the same regions (discontinuous, not preferentially accumulated at exons as usually happens due to co-transcriptional splicing) and their physical proximity (Fig 2), I wonder whether, instead of replication fork pausing/stalling events as the authors claim, BrdU peaks might account for initiation events distinct from canonical replication initiation sites (that, at transcribed regions might be RNA-primed). One way to test this will be by analyzing the location of BrdU peaks relative to high-resolution Repli-seq regions (like in Zhao et al 2020 Genome Biol 21: 76).

*Reply: This is an interesting point raised by the reviewer and we have looked into this more carefully. We have obtained from the Gilbert lab the list of the initiation and termination zones they identified in their Zhao et al. paper. Regarding the initiation zones, they have identified a total of 2371 initiation zones in HCT116 cells, with an average median width of 50kb. In the figure below there are a series of snapshots with in blue the Repli-Seq data from Zhao et al., in black the locations of our BrdU peaks, in red initiation zones from Zhao et al. and in green termination zones. Overall, this shows that BrdU peaks and initiation zones do not specifically overlap, and performing an overlap analysis we found that 9.3% of our BrdU peaks overlap with these initiation zones, while 26.6% overlap with termination sites.*

*Snapshot across multiple genomic large regions of Repli-Seq files from Zhao et al. in blue, black bars indicate the location of our BrdU peaks, red and light blue bars indicate the location of the initiation and termination zones from Zhao et al.*

*Reply: Because this data did not assess the overlap of our BrdU peaks with non-canonical or stochastic origins, we performed an additional analysis of origins of replication identified in the Akerman et al. paper. Akerman et al. identified core origins, that are more ubiquitously used across multiple cell lines, and stochastic origins, used in fewer cell lines and/or cell line specific. We did assess the frequency of overlap and distance of our BrdU peaks with both core and stochastic origins (Fig S1F), using the same parameters of Guilbaud et al, NAR 2022. We analyzed also whether these overlapping sites where active as origins in our cells, beyond overlapping with the same genomic sites. To do this we analyzed i) our PCNA ChIP-Seq levels at origins identified in multiple studies overlapping with our BrdU peaks (Fig S1G), and ii) whether we could identify a split in Okazaki fragments expected at origins between Watson and Crick strands (Fig S1H). Neither of these analyses showed any clear indication that even the BrdU peaks that overlap with regions previously identified as origins of replication, are actually active origins of replication in our cell line. There are definitely some cases where BrdU peaks might overlap with origins, but for the very vast majority they are not related to origins of replication.*

Another possibility fitting with the rest of the observations shown in the manuscript is that BrdU peaks represent preferential sites of repair synthesis after DNA damage, independent of transcription. The authors should consider and test other possibilities for interpreting their data.

*Reply: This is an extremely valid point that we hadn’t considered, hence thanks to the reviewer for this suggestion. We have performed this analysis and have included it in this revised version of the manuscript (Fig S1B). To address this point, we have identified hotspot sites of gH2AX in our ChIP-Seq, performing a peak calling analysis as we did for the BrdU-Seq. We have identified overall 44682 gH2AX peaks in our dataset, where we have much higher gH2AX levels than at the BrdU peaks. At the same time, the BrdU levels at gH2AX peaks are much lower than those at BrdU peaks. Therefore, while we can’t exclude that some of the BrdU accumulation at BrdU peaks is perhaps consequence of DNA damage repair synthesis, the high levels of BrdU at BrdU peaks are not solely dependent on DNA damage repair synthesis.*

As mentioned above, I also do not understand what the short RNA reads from the Chr-RNA-seq experiments represent since they do not cover the whole gene body nor accumulate at exons like chromatin-bound RNA reads do (Fig 2). Could the authors show the TSS-centered read coverage of the corresponding genes from panels 2B and 2E?

*Reply: We are showing below the Chr-RNA-Seq signal centered around the TSS for genes replicated in the first (A) and the second (C) timepoints. The procedure we use to get the chromatin bound RNA is the one developed by the Churchman lab for their NET-Seq (Mayer et al., Cell 2015). The signal is very strong at the first exon as expected in a Chr-RNA-Seq, much higher for genes replicated in the first timepoint than in the later timepoints as we previously shown (Wang et al., Cell Reports 2021). However, this procedure allows us still to recover a relatively high coverage over introns, as it is possible to appreciate from the metagene profiles at 5’- and 3’-ends of exons of genes replicated in the first timepoint. The coverage over the introns is specifically representing the nascent RNA produced in the cell, that is only transient and short-lived. As such, those accumulations of RNA signal around the BrdU peaks are representing sites of accumulation of the RNA Pol II while transcribing, hence sites where it stalls or pauses. Regarding the signal over exons however we can’t be completely sure as they are generally co-transcriptionally spliced, with RNA Pol II perhaps many kb away from where the signal is coming from. This is also why when we present representative peaks, we show those that are in introns and not those that overlap with exons, as transcription and replication signals could be unrelated there.*

A) *Metagene profile for Chr-RNA-Seq signal at TSS +/- 1kb for genes replicated in the first timepoint, separated in codirectional, head on and transition. B) Metagene profile for Chr-RNA-Seq at the 5’-end and the 3’-end of exons of genes replicated in the first timepoint. C) As for A) only for genes replicated in the second timepoint.*

Other points:

1.      The message of the paper is built around the impact of RNAPII transcription on DNA replication, however only 38% of the BrdU peaks are in genes. Since their association with fragile sites and breakpoints is indistinguishable regardless they are genic or not, and considering other potential explanations for their origin, I think the authors should consider changing the focus of the text (and title).

*Reply: The title of the manuscript has now been changed to accommodate for the overall broader message of the manuscript that is not only transcription centered.*

2.      Regarding the above, the authors correlate the BrdU peaks with multiple published datasets (Fig 1F, 1G, S1, S2). In all cases, the cell lines from which the data were derived should be clarified in the text. This is especially relevant in the case of CFS, as they are cell type specific (Michelle Debatisse´s work).

*Reply: This information has now been included in the main text.*

3.      Relevant controls to support their claim that BrdU peaks are indeed hotspots of DNA damage would be showing BrdU-seq, P-H2AX-seq and GLOE-seq signals at TSS in Fig 1 BRCA1, BRCA2, FANCD2 and RAD51 in Fig S1.

*Reply: These have now been included and they overall show that signals for these factors at TSS of long genes compared to BrdU peaks are different. The only exceptions are BRCA1 and BRCA2, as these have been previously shown to have roles in regulating RNA Pol II transcription (Gardini et al, EMBO J 2014; Gruber et al., Cell Reports 2019).*

4.      Why did the authors use S5P-RNAPII instead of S2P-RNAPII for the PLA experiments in Fig 3A? Wouldn´t the latter be more informative of the co-existence of on-going replication and transcription?

*Reply: While S2P-RNAPII is generally referred as the elongating form of RNAPII, its levels increase during elongation but are low at towards the beginning of a gene (Zabrowska et al., NSMB 2016). S5P-RNAPII is present instead from the beginning of transcription, hence we have chosen S5P instead of S2P to get information across the whole length of the gene. Indeed, in a recent publication where they have done PLA with EdU vs S2P and EdU vs S5P, they find more PLA dots in the S5P compared to the S2P (Fenstermarker et al., Nature 2013), hence the S5P is simply more sensitive.*

5.      In Fig 3C the maximum BrdU signal occurs at the TSS of genes in cells treated with transcription inhibitors. DMSO unnormalized signal should be also shown in these metagenes (see also my comment in point 3) to better understand what we are dealing with here.

*Reply: Below are the unnormalized BrdU-Seq profiles in DMSO, DRB and alpha-amanitin for genes of different length. The sharp peak at the TSS in the normalized profiles is because upon treatment with the two transcription inhibitors there is slightly less pronounced gap at the TSS. We can replace the normalized profile with the ones below in the figure. Because metagene profiles present an average signal change between DMSO and transcription inhibitors, we presented in the manuscript the gene-to-gene fold changes, as these allow to determine whether any effect is restricted to few genes or is global. As we have 2 repeats for each condition, the BrdU-Seq signal for each condition is the average of the 2 repeats.*

6.      In lines 549-551 the authors conclude that "changes in RNAPII elongation directly affect DNA replication progression and/or efficiency, with a direct correlation to genome instability levels in genes". To claim a direct correlation, they should perform a pair-wise comparison between BrdU-seq reads and P-H2AX reads at the same genes.

*Reply: We have performed the analysis suggested by the reviewer, correlating BrdU-Seq levels on genes replicated in the first timepoint with gH2AX ChIP-Seq levels across the same genes. We found an overall positive correlation of 0.43 between BrdU and gH2AX levels inside the same gene (p-value < 10^-4^) across all transcribed genes. If we separate genes by their transcription levels in RPKM in low, medium and high, the correlations are 0.333, 0.421 and 0.566, all p-value < 10^-4^. This supports that the levels of DNA damage and how replication progresses through a gene, are related to the transcription activity in the gene. We have not included this result in the manuscript and this section has been changed in the manuscript, but are happy to include this result if the reviewer thinks it is an important point.*

7.      The analyses of PCNA coverage along genes of different lengths is intriguing. How to reconcile the clear decrease in PCNA signal along genes >100 kb indicative of faster moving forks with an increase in BrdU signal (Fig 3A-B)?

*Reply: The decrease in PCNA signal at long genes could have been the consequence of two possible scenarios:*

1) *Replication forks do not progress efficiently inside transcribed genes;*
2) *Replication forks progress inside transcribed genes, but their speed changes and this alters the density of replication fork components.*

*The fact that we observe DNA synthesis across these genes indicated by the BrdU-Seq signal, excludes scenario 1 and indicates that a change in replication forks density and distribution along the genes, is indicative of a change in replication speed.*

*Regarding the BrdU-Seq signal that slightly increases inside long genes, that is likely linked to the fact that BrdU peaks are enriched in longer genes, and this may contribute to the average higher signal for BrdU inside genes.*

8.      Fig 5E; how the analysis is performed needs clarification in the text and methods.

*Reply: Additional information on how the experiment was performed has now been included in the results and methods sections.*

Reviewer #2:

Rojas et al present work addressing important issues about replication and transcription machines cohabiting in genic regions. They use whole genome approaches analysing nascent replication and transcription products as well as ChIP to correlate replication, transcription and DNA damage, mainly. Their conclusions are that ongoing transcription and replication co-exist. Pausing of the two machineries coincide.  Active transcription may accelerate replication due to lowering nucleosome density. Pausing sites correlate with several signs of DNA damage and genome re-arrangements. The prevention of re-arrangements requires DNA damage signalling.

The argumentation line of the manuscript is hard to follow. The manuscript is complicated and lengthy and the language is imprecise. Whilst the first two figures (including 3a) seem relatively clear, with sufficiently strong experimental effects measured, the manuscript becomes increasingly unclear from this point. Partly, effects measured seem minute, for example RNAi experiments in Fig 3. Moreover, similar experiments seem to be displayed in different ways, which makes it even more difficult to follow. Furthermore, it remains often unclear to me what the authors intend to state with specific experiments. Conclusions are not clearly formulated.

*Reply: We apologize that our manuscript was not clear, and we recognize the limitations with some of the previously presented data, where some differences were small and convoluted in their analysis. We have now restructured the manuscript, changed the order of the figures and brought forward any hypothesis that we wanted to assess. Hopefully, this is making the manuscript more linear and easier to follow.*

Sometimes it remains unclear to me what the power of particular experimental techniques are, particularly in light of weak effects measured. I appreciate that averaging effects over the whole genome (or all genes) dilutes effects on individual genes, but lends strength to the experiments by their statistical power. However, this finds its limits when effect size starts to affect the credibility of conclusions. A mixture of two measures could lend strength on the experimental results:
1) providing data on the power of experiments by creating clearly defined predictable situations. For example, aphidicolin slows replication predictably and tunably. DNA damage can be induced to test the effect on transcription and replication.
2) complementing whole genome analyses with analyses of specific genes. This could lead to stronger effects. Ideally, the authors observe tunable genes (endogenous or a tet-inducible transgene, for example) that they could switch on and off and analyse the consequences. Manipulation is tried in figure 2, which is appreciated. But the effects were small (RNAi) and not gene-specific but systemic.

*Reply: We thank the reviewer for this suggestion as it prompted us to analyze our data in a different way. We did consider the option of an inducible transgene initially. However, previous work showed that a single gene being transcribed can alter the replication timing of a broad region (Koren et al., Cell 2014). Therefore, we were concerned about the impact of an inducible system on replication timing, and therefore how and when to analyze the impact of transcription on replication progression. At the same time, replication stress inducing agents like aphidicolin can impact gene transcription too (Mazouzi et al., Cell Reports 2016), as well as the induction of DNA damage in a gene or globally (Narayanan et al., Scientific Reports 2017; Williamson et al., Cell 2017). This would make it difficult to separate the impact of the replication stress and/or DNA damage response from the direct impact of stalled replication forks and DNA damage on replication and transcription inside a gene.*

*However, as we used multiple drug treatments in our manuscript (DRB, alpha-amanitin, ATM inhibitor and ATR inhibitor), we analyzed the impact of the changes in gene expression induced by these inhibitors, on BrdU peaks specifically inside these genes. We are now able to present many single BrdU peaks examples in genes that are either up- or down-regulated following treatments with the aforementioned drugs, showing how the BrdU levels change accordingly to the changes in gene expression. The analysis was restricted to genes replicated in the first timepoint, as that was when we performed the BrdU-Seq. The only case we could not assess in detail was the ATM inhibitor, as this seems to induce only little changes to gene expression in the absence of external insults, none affecting a gene where a BrdU peak was found (Corti et al., Scientific Reports 2019).*

**Second round of review**

**Reviewer 1**

The authors have addressed all my previous concerns and have done a great job in re-structuring the whole manuscript, that now is more solid and reads better. I believe the work will be of interest in several fields.

I still do recommend some minor polishing actions:

1) Some sentences, including the new title, will benefit for a native english editing.
2) The quality of the main figures needs to be improved. Particularly, the scale of the metaplots is invisible in most figures.
3) There are some mistakes in the labeling of the figure panels, ie Fig S3F. Please, revise all.

**Authors Response**

**Point-by-point responses to the reviewers’ comments:**

Reviewer #1:

The authors have addressed all my previous concerns and have done a great job in re-structuring the whole manuscript, that now is more solid and reads better. I believe the work will be of interest in several fields.

I still do recommend some minor polishing actions:

1) Some sentences, including the new title, will benefit for a native english editing.
2) The quality of the main figures needs to be improved. Particularly, the scale of the metaplots is invisible in most figures.
3) There are some mistakes in the labeling of the figure panels, ie Fig S3F. Please, revise all.

*Reply: Regarding Reviewer's 1 comments, the manuscript has been totally edited, the quality of the figures has been improved with higher resolution pdf files and mistakes in the labelling corrected.*
